# Supplementary material for: Inhibitory activity of traditional plants against Mycobacterium smegmatis and their action on Filamenting temperature sensitive mutant Z (FtsZ)—A cell division protein
Source: PLoS One. 2020 May 1;15(5):e0232482. doi: 10.1371/journal.pone.0232482 (PMC7195194; doi:10.1371/journal.pone.0232482)
Supplement: S1 Table — (DOCX) [file pone.0232482.s001.docx]

**Table 1S. GTPase IC_50_ of the HXM plant extracts**

| **Name of the plant / Compound** | **Common Name** |  | **GTPase IC_50_** |
| --- | --- | --- | --- |
| *Acacia nilotica* | Babool (Leaves) |  | 1.399 (mg/mL) |
| *Aegle marmelos* | Bael/Vilvam (Unriped fruit pulp) |  | 1.329 (mg/mL) |
| *Glycyrrhiza glabra* | Licorice  (Dried Root) |  | 1.564 (mg/mL) |
| D-Pinitol | - |  | 1.524 (µg/mL) |
| Berberine | - |  | 1.861 (µg/mL) |
