# Supplementary material for: Inhibitory activity of traditional plants against Mycobacterium smegmatis and their action on Filamenting temperature sensitive mutant Z (FtsZ)—A cell division protein
Source: PLoS One. 2020 May 1;15(5):e0232482. doi: 10.1371/journal.pone.0232482 (PMC7195194; doi:10.1371/journal.pone.0232482)
Supplement: S2 Table — (DOCX) [file pone.0232482.s002.docx]

**Table 2S. Effect of HXM extracts / D-Pinitol on cell elongation of *M. smegmatis***

| **Plant extracts / Compound** | **Length of Cell elongation (µm)** |
| --- | --- |
| Untreated Cells | 3.9 ± 0.5 |
| Isoniazid | 9.7 ± 0.6 |
| Rifampicin | 11.5 ± 0.2 |
| *A. nilotica* | 8.2 ± 1.0 |
| *A.marmelos* | 15.7 ± 1.2 |
| *G. glabra* | 13.5 ± 0.5 |
| D-Pinitol | 12.7 ± 0.1 |
