# Supplementary material for: Inhibitory activity of traditional plants against Mycobacterium smegmatis and their action on Filamenting temperature sensitive mutant Z (FtsZ)—A cell division protein
Source: PLoS One. 2020 May 1;15(5):e0232482. doi: 10.1371/journal.pone.0232482 (PMC7195194; doi:10.1371/journal.pone.0232482)
Supplement: S3 Table — (DOCX) [file pone.0232482.s003.docx]

**Table 3S. List of primers used in the FtsZ gene expression study**

|  | **Gene** | **Primers** | |
| --- | --- | --- | --- |
| Reference Gene | *Mycobacterium*16srRNA rrsB | FP | **5'’-**AGTCTGGGCCGTATCTCAGT- **3’** |
|  |  | RP | **5'’-**CTGCCCTGCACTTTGGGATA- **3’** |
| Gene of Interest | *M.smegmatis* –Ftsz | FP | **5'’-**CTCCTCGATGTCGTCCTTGG - **3’** |
|  |  | RP | **5'’-**TCAAGGTGGTTGGTATCGGC - **3’** |
