# Supplementary material for: Inhibitory activity of traditional plants against Mycobacterium smegmatis and their action on Filamenting temperature sensitive mutant Z (FtsZ)—A cell division protein
Source: PLoS One. 2020 May 1;15(5):e0232482. doi: 10.1371/journal.pone.0232482 (PMC7195194; doi:10.1371/journal.pone.0232482)
Supplement: S4 Table — (DOCX) [file pone.0232482.s004.docx]

**Table 4S. Relative expression levels of FtsZ**

| **Plant extracts** | **16sRNA Control rrsB** | **Gene Of Interest FtsZ** | **ΔCt** | **ΔΔCt** | **2^(ΔΔCt) Fold expression** | **SD** |
| --- | --- | --- | --- | --- | --- | --- |
| Untreated cells | 16.84 | 26.57 | 9.73 | 0 | 1 | 0 |
| *Acacia nilotica* | 16.53 | 25.45 | 8.92 | -1.11 | 2.15 | 0.26 |
| *Aegle marmelos* | 16.71 | 24.7 | 7.99 | -1.74 | 3.34 | 0.32 |
| *Glycyrrhiza glabra* | 16.42 | 24.06 | 7.64 | -2.09 | 4.25 | 0.19 |
| D - Pinitol | 16.92 | 25.16 | 8.24 | -1.49 | 2.80 | 0.31 |
