# Supplementary figures and images for: Inhibitory activity of traditional plants against Mycobacterium smegmatis and their action on Filamenting temperature sensitive mutant Z (FtsZ)—A cell division protein
Source: PLoS One. 2020 May 1;15(5):e0232482. doi: 10.1371/journal.pone.0232482 (PMC7195194; doi:10.1371/journal.pone.0232482)

**Figure 1S. GCMS peaks and table values  *of A.nilotica***
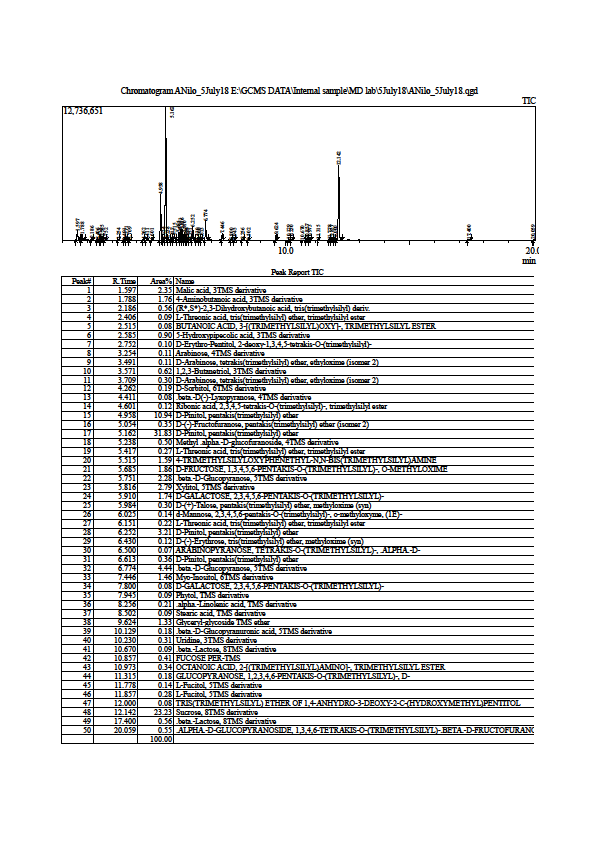

Supplement: S1 Fig — (DOCX) [file pone.0232482.s005.docx]

**Figure 2S. GCMS peaks and table values  *of A.marmelos***


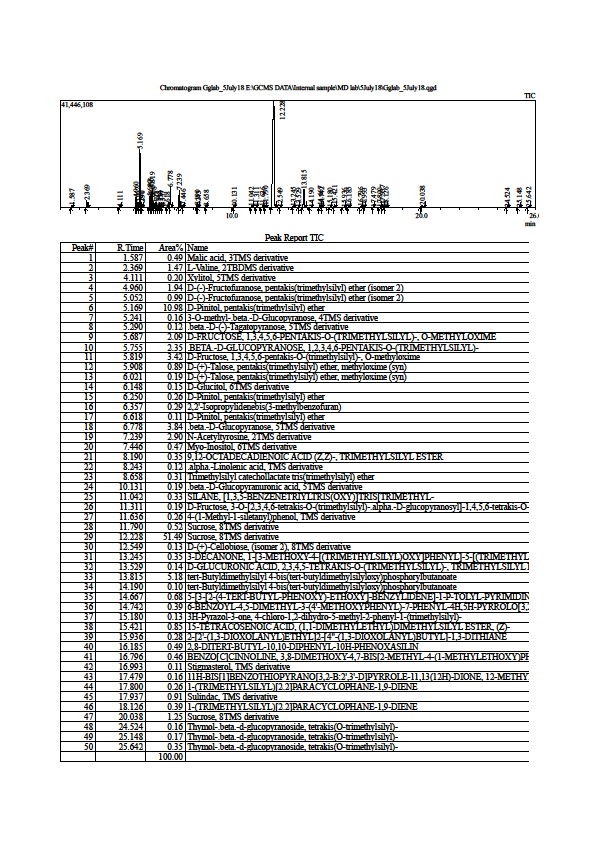

Supplement: S2 Fig — (DOCX) [file pone.0232482.s006.docx]

**Figure 3S. GCMS peaks and table values  *of G.glabra***


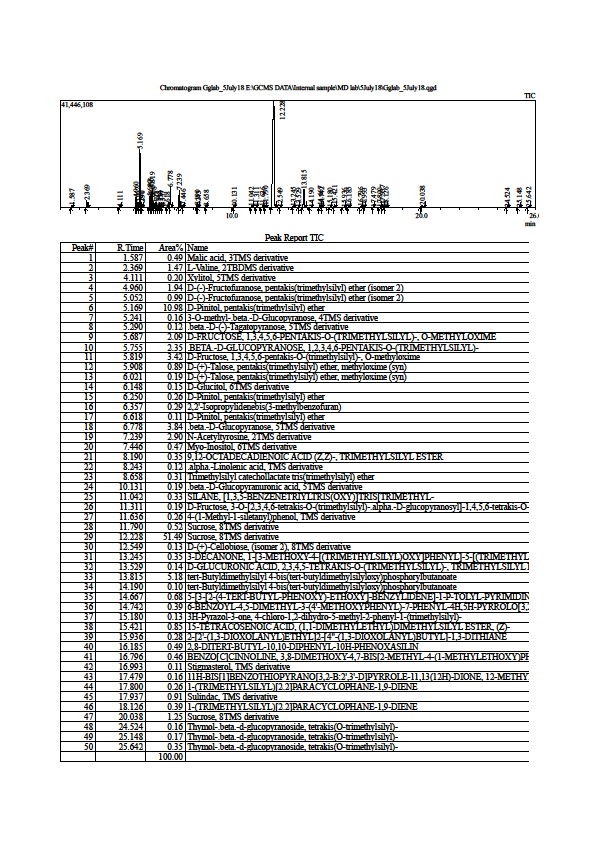

Supplement: S3 Fig — (DOCX) [file pone.0232482.s007.docx]

**Figure 7S. HPLC of *A. nilotica***

**
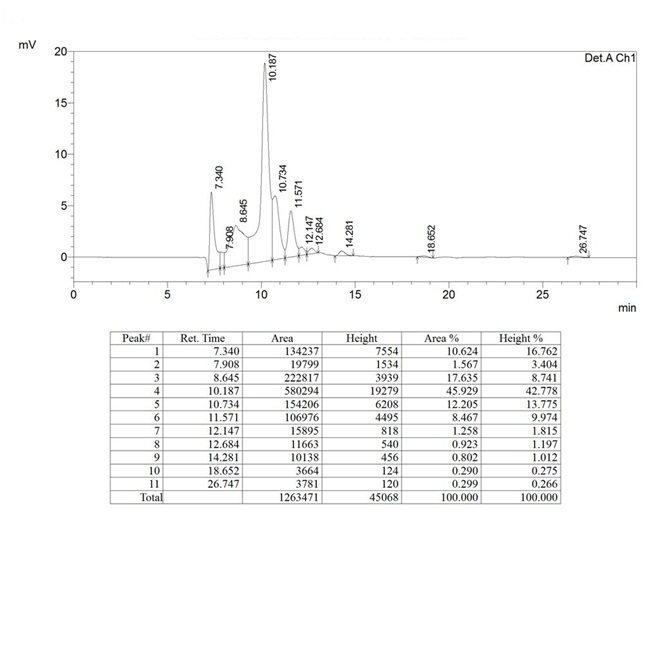
**

Supplement: S7 Fig — (DOCX) [file pone.0232482.s011.docx]

**Figure 8S. HPLC of *A. marmelos***

**
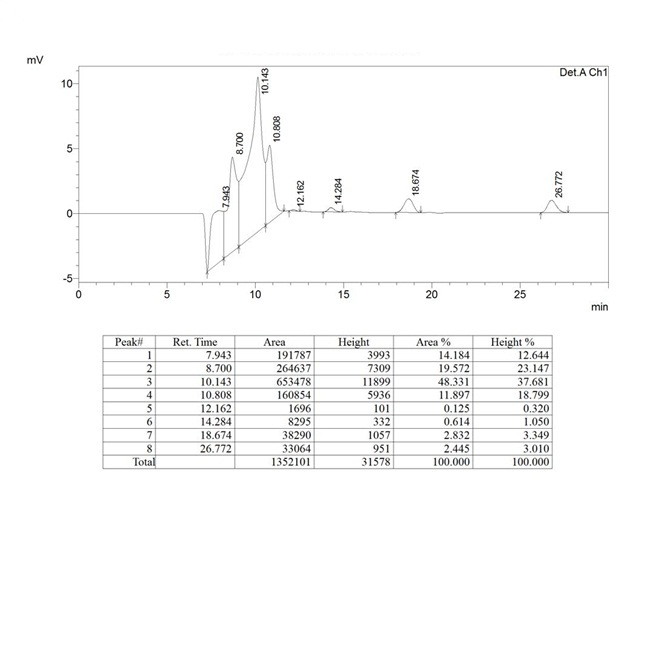
**

Supplement: S8 Fig — (DOCX) [file pone.0232482.s012.docx]

**Figure 9S. HPLC of *G. glabra***

**
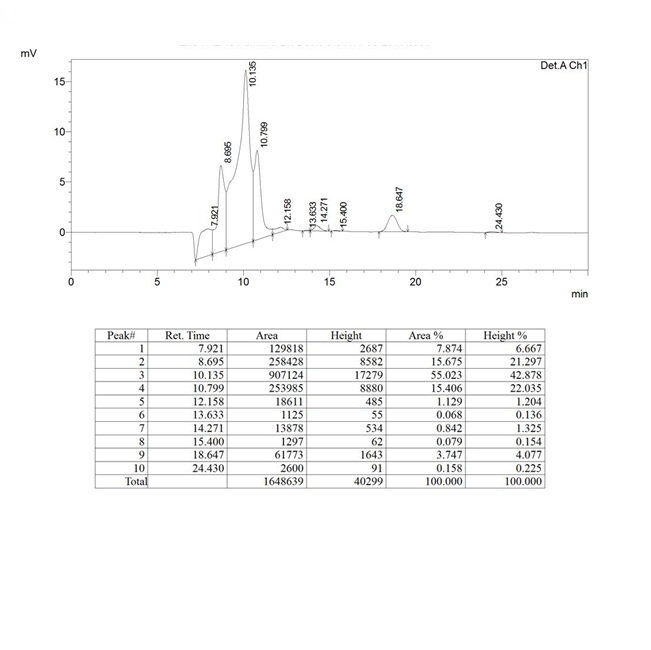
**

Supplement: S9 Fig — (DOCX) [file pone.0232482.s013.docx]

**Figure 10S. HPLC of D- Pinitol (Standard)**

**
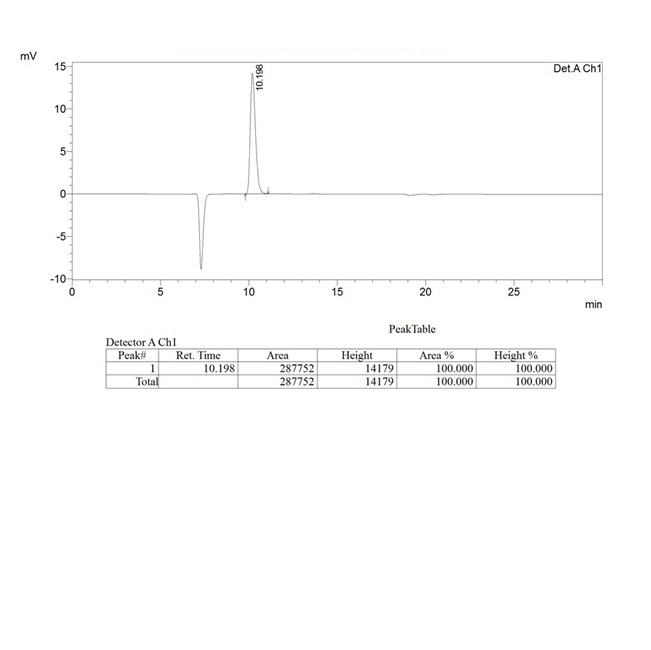
**

Supplement: S10 Fig — (DOCX) [file pone.0232482.s014.docx]
