# Supplementary material for: Inhibitory activity of traditional plants against Mycobacterium smegmatis and their action on Filamenting temperature sensitive mutant Z (FtsZ)—A cell division protein
Source: PLoS One. 2020 May 1;15(5):e0232482. doi: 10.1371/journal.pone.0232482 (PMC7195194; doi:10.1371/journal.pone.0232482)

**Figure 4S. Mass fragmentation of *A.nilotica***


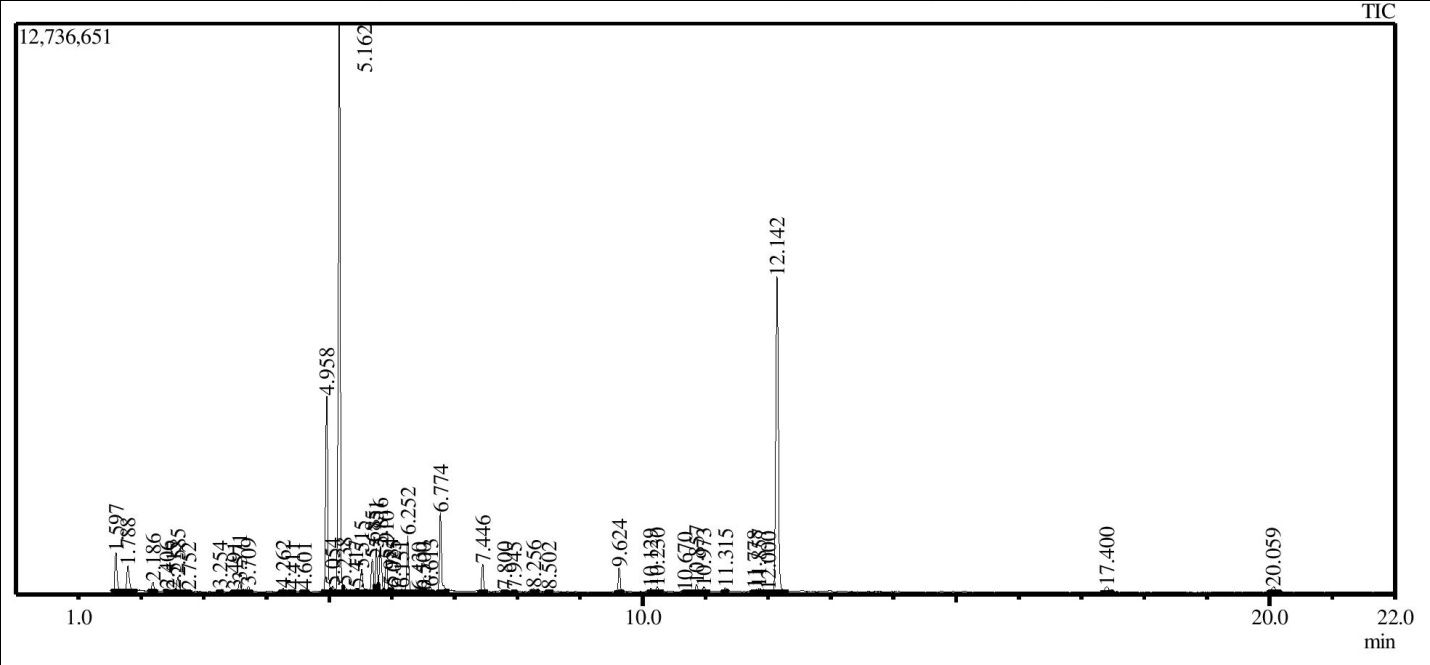


R.Time:5.160(Scan#:733) MassPeaks:614

RawMode:Averaged 5.155-5.165(732-734) BasePeak:73.05(1303518)

BG Mode:Calc. from Peak Group 1 - Event 1


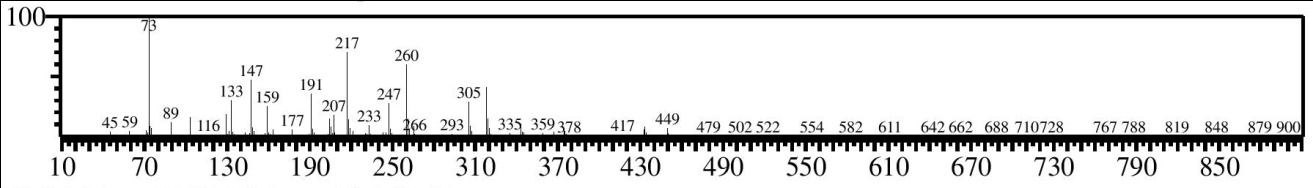


Entry:237540 Library:NIST14.lib

SI:90 Formula:C22H54O6Si5 CAS:0-00-0 MolWeight:554 RetIndex:2085

CompName:D-Pinitol, pentakis(trimethylsilyl) ether


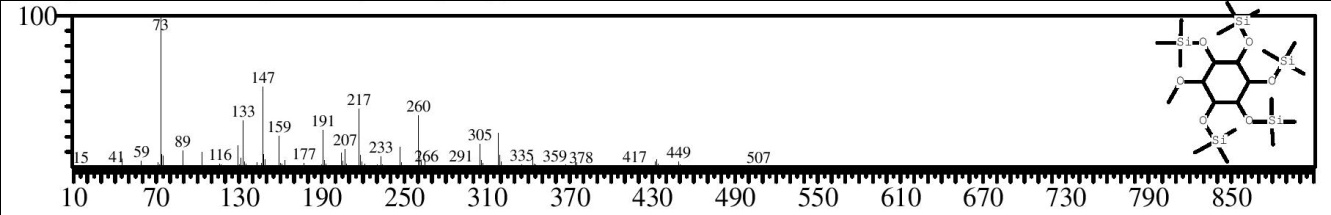

Supplement: S4 Fig — (DOCX) [file pone.0232482.s008.docx]
