# Supplementary material for: Inhibitory activity of traditional plants against Mycobacterium smegmatis and their action on Filamenting temperature sensitive mutant Z (FtsZ)—A cell division protein
Source: PLoS One. 2020 May 1;15(5):e0232482. doi: 10.1371/journal.pone.0232482 (PMC7195194; doi:10.1371/journal.pone.0232482)
Supplement: S11 Fig — (DOCX) [file pone.0232482.s015.docx]

**Figure 11S.** Time Kill curves of Isoniazid in combination with HXM extracts of a) *A. nilotica*, b) *A. marmelos*, and c) *G. glabra* against *M. smegmatis*

**
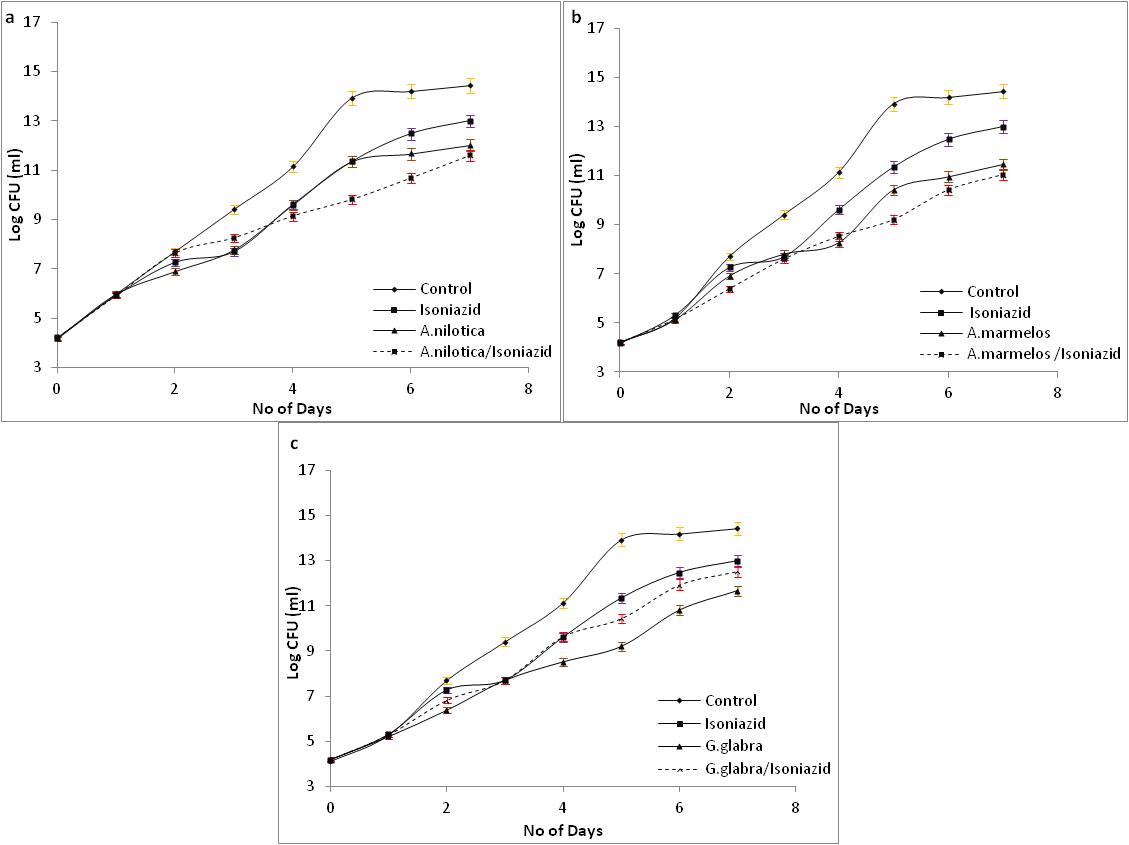
**
