# Supplementary material for: Inhibitory activity of traditional plants against Mycobacterium smegmatis and their action on Filamenting temperature sensitive mutant Z (FtsZ)—A cell division protein
Source: PLoS One. 2020 May 1;15(5):e0232482. doi: 10.1371/journal.pone.0232482 (PMC7195194; doi:10.1371/journal.pone.0232482)
Supplement: Attachment — Submitted filename: PONE-D-19-29098_reviewer_1.pdf [file pone.0232482.s017.pdf]

PLOS ONE

Antitubercular activity of plant extracts and their action on cell division protein (FtsZ)

--Manuscript Draft--

**Manuscript Number:**	PONE-D-19-29098
**Article Type:**	Research Article
**Full Title:**	Antitubercular activity of plant extracts and their action on cell division protein (FtsZ)
**Short Title:**	Antitubercular activity and role in cell division
**Corresponding Author:**	Radhika Ravindran<br>Indian Institute of Technology Madras<br>Chennai, Tamil Nadu INDIA
**Keywords:**	Time kill curve, DPPH, Cell elongation, Bac light assay, GTPase activity, Light scattering, q PCR.
**Abstract:**	To assess whether plant extracts / phytochemical (D-Pinitol) synergistically combine with anti-tubercular drugs and act on M. smegmatis as well as assess their mode of action on M.tb Filamenting temperature sensitive mutant Z (FtsZ) protein.Resazurin microtitre plate assay (Checker board) was performed to analyze the activity of plant extracts against M. smegmatis . Synergistic behaviour of plant extracts / D-Pinitol with Isoniazid (INH) and Rifampicin were determined by time–kill and checker board assays. Elongation of M. smegmatis cells due to this treatment was determined by light microscopy. The effect of HXM plant extracts on cell viability was determined using PI/SYTO9 dual dye reporter Live/Dead assay. Action of HXM plant extracts / D-Pinitol on inhibition of FtsZ protein was done using GTPase light scattering assay and quantitative Polymerase Chain Reaction (qPCR).The Hexame-methanolic plant extract of Acacia nilotica, Aegle marmelos and Glycyrrhiza glabra showed anti-mycobacterial activity at 1.56 ± 0.03, 1.32 ± 0.02 and 1.25 ± 0.03 mg/ml respectively and that of Isoniazid and Rifampicin were 29 ± 0.14 μM and 2.43 ± 0.04 μM respectively. These plant extracts and major phytochemical exudate D-Pinitol was found to act synergistically with anti-mycobacterial drugs Rifampicin and isoniazid with an FIC index ~ 0.20. Time-Kill kinetics studies indicates that, these plant extracts were bacteriostatic in nature. D-Pinitol in conjunction with RIF and INH exhibited a 2 Log retardation in the growth of viable cells compared to untreated. Attempt to elucidate their mode of action through phenotypic analysis indicated that these plant extracts and D-Pinitol was found to interfere in cell division there by leading to an abnormal elongated cellular morphology.HXM extracts and D-Pinitol synergistically combined with the first line tuberculosis drugs, INH and RIF, to act on M. smegmatis . The increase in the length of M. smegmatis cells on treatment with D-Pinitol and HXM extract of the plants indicated that they hinder the cell division mechanism thereby leading to a filamentous phenotype, and finally leading to cell death. In addition, the integrity of the bacterial cell membrane is also altered causing cell death. Further gene expression analysis showed that these plant extracts and D-Pinitol hampers with function of Filamenting temperature sensitive-Z (FtsZ) protein which was confirmed through in-vitro inhibition FtsZ –GTPase enzymatic activity.
**Order of Authors:**	Radhika Ravindran
	Kartik Mitra
	Gayathri Chakrapani
	Mukesh Doble
**Additional Information:**	
**Question**	**Response**
**Financial Disclosure**<br><br>Enter a financial disclosure statement that describes the sources of funding for the work included in this submission. Review	Funding is not received

Powered by Editorial Manager® and ProduXion Manager® from Aries Systems Corporation

the submission guidelines for detailed requirements. View published research articles from *PLOS ONE* for specific examples.<br><br>This statement is required for submission and **will appear in the published article** if the submission is accepted. Please make sure it is accurate.<br><br>**Unfunded studies**<br>Enter: *The author(s) received no specific funding for this work.*<br><br>**Funded studies**<br>Enter a statement with the following details:<br>• Initials of the authors who received each award<br>• Grant numbers awarded to each author<br>• The full name of each funder<br>• URL of each funder website<br>• Did the sponsors or funders play any role in the study design, data collection and analysis, decision to publish, or preparation of the manuscript?<br>• **NO** - Include this sentence at the end of your statement: *The funders had no role in study design, data collection and analysis, decision to publish, or preparation of the manuscript.*<br>• **YES** - Specify the role(s) played.<br><br>* typeset	
**Competing Interests**<br><br>Use the instructions below to enter a competing interest statement for this submission. On behalf of all authors, disclose any competing interests that could be perceived to bias this work—acknowledging all financial support and any other relevant financial or non-financial competing interests.<br><br>This statement **will appear in the published article** if the submission is accepted. Please make sure it is accurate. View published research articles from *PLOS ONE* for specific examples.	The authors have declared that no competing interests exist.

**NO authors have competing interests**<br><br>Enter: *The authors have declared that no competing interests exist.*<br><br>**Authors with competing interests**<br><br>Enter competing interest details beginning with this statement:<br><br>*I have read the journal's policy and the authors of this manuscript have the following competing interests: [insert competing interests here]*<br><br>* typeset	
**Ethics Statement**<br><br>Enter an ethics statement for this submission. This statement is required if the study involved:<br><br>• Human participants<br>• Human specimens or tissue<br>• Vertebrate animals or cephalopods<br>• Vertebrate embryos or tissues<br>• Field research<br><br>Write "N/A" if the submission does not require an ethics statement.<br><br>General guidance is provided below. Consult the submission guidelines for detailed instructions. **Make sure that all information entered here is included in the Methods section of the manuscript.**	yes,<br>Indian institute of technology, Madras

Powered by Editorial Manager® and ProduXion Manager® from Aries Systems Corporation

**Format for specific study types**<br><br>**Human Subject Research (involving human participants and/or tissue)**<br>• Give the name of the institutional review board or ethics committee that approved the study<br>• Include the approval number and/or a statement indicating approval of this research<br>• Indicate the form of consent obtained (written/oral) or the reason that consent was not obtained (e.g. the data were analyzed anonymously)<br><br>**Animal Research (involving vertebrate animals, embryos or tissues)**<br>• Provide the name of the Institutional Animal Care and Use Committee (IACUC) or other relevant ethics board that reviewed the study protocol, and indicate whether they approved this research or granted a formal waiver of ethical approval<br>• Include an approval number if one was obtained<br>• If the study involved *non-human primates*, add *additional details* about animal welfare and steps taken to ameliorate suffering<br>• If anesthesia, euthanasia, or any kind of animal sacrifice is part of the study, include briefly which substances and/or methods were applied<br><br>**Field Research**<br><br>Include the following details if this study involves the collection of plant, animal, or other materials from a natural setting:<br>• Field permit number<br>• Name of the institution or relevant body that granted permission	
**Data Availability**<br><br>Authors are required to make all data underlying the findings described fully available, without restriction, and from the time of publication. PLOS allows rare exceptions to address legal and ethical concerns. See the PLOS Data Policy and FAQ for detailed information.	Yes - all data are fully available without restriction

Powered by Editorial Manager® and ProduXion Manager® from Aries Systems Corporation

A Data Availability Statement describing where the data can be found is required at submission. Your answers to this question constitute the Data Availability Statement and **will be published in the article**, if accepted.<br><br>**Important:** Stating 'data available on request from the author' is not sufficient. If your data are only available upon request, select 'No' for the first question and explain your exceptional situation in the text box.<br><br>Do the authors confirm that all data underlying the findings described in their manuscript are fully available without restriction?	
**Describe where the data may be found in full sentences. If you are copying our sample text, replace any instances of XXX with the appropriate details.**<br><br>• If the data are **held or will be held in a public repository**, include URLs, accession numbers or DOIs. If this information will only be available after acceptance, indicate this by ticking the box below. For example: *All XXX files are available from the XXX database (accession number(s) XXX, XXX.).*<br>• If the data are all contained **within the manuscript and/or Supporting Information files**, enter the following: *All relevant data are within the manuscript and its Supporting Information files.*<br>• If neither of these applies but you are able to provide **details of access elsewhere**, with or without limitations, please do so. For example:<br><br>*Data cannot be shared publicly because of [XXX]. Data are available from the XXX Institutional Data Access / Ethics Committee (contact via XXX) for researchers who meet the criteria for access to confidential data.*<br><br>*The data underlying the results presented in the study are available from (include the name of the third party*	Data cannot be shared publicly

and contact information or URL).<br>• This text is appropriate if the data are owned by a third party and authors do not have permission to share the data.<br><br>* typeset	
Additional data availability information:	Tick here if the URLs/accession numbers/DOIs will be available only after acceptance of the manuscript for publication so that we can ensure their inclusion before publication.

Manuscript
Click here to download Manuscript PLOS.docx

Antitubercular activity of plant extracts and their action on cell division protein (FtsZ)

Radhika Ravindran, Gayathri Chakrapani, Kartik Mitra and Mukesh Doble*

Bioengineering and Drug Design Lab, Department of Biotechnology, Indian Institute of Technology, Madras, 600036, India.

*** Corresponding author**

Tel.: +91- 44 2257 4107; Fax: +91 44 2257 4102.

E-mail address: mukeshd@iitm.ac.in

Abstract

To assess whether plant extracts / phytochemical (D-Pinitol) synergistically combine with anti-tubercular drugs and act on *M. smegmatis* as well as assess their mode of action on *M.tb* Filamenting temperature sensitive mutant Z (FtsZ) protein.Resazurin microtitre plate assay (Checker board) was performed to analyze the activity of plant extracts against *M. smegmatis*. Synergistic behaviour of plant extracts / D-Pinitol with Isoniazid (INH) and Rifampicin were determined by time–kill and checker board assays. Elongation of *M. smegmatis* cells due to this treatment was determined by light microscopy. The effect of HXM plant extracts on cell viability was determined using PI/SYTO9 dual dye reporter Live/Dead assay. Action of HXM plant extracts / D-Pinitol on inhibition of FtsZ protein was done using GTPase light scattering assay and quantitative Polymerase Chain Reaction (qPCR).The Hexame-methanolic plant extract of *Acacia nilotica*, *Aegle marmelos* and *Glycyrrhiza glabra* showed anti-mycobacterial activity at 1.56 ± 0.03, 1.32 ± 0.02 and 1.25 ± 0.03 mg/ml respectively and that of Isoniazid and Rifampicin were 29 ± 0.14 µM and 2.43 ± 0.04 µM respectively. These plant extracts and major phytochemical exudate D-Pinitol was found to act synergistically with anti-mycobacterial drugs Rifampicin and isoniazid with an FIC index ~ 0.20. Time-Kill kinetics studies indicates that, these plant extracts were bacteriostatic in nature. D-Pinitol in conjunction with RIF and INH exhibited a 2 Log retardation in the growth of viable cells compared to untreated. Attempt to

elucidate their mode of action through phenotypic analysis indicated that these plant extracts and D-Pinitol was found to interfere in cell division there by leading to an abnormal elongated cellular morphology.HXM extracts and D-Pinitol synergistically combined with the first line tuberculosis drugs, INH and RIF, to act on *M. smegmatis*. The increase in the length of *M. smegmatis* cells on treatment with D-Pinitol and HXM extract of the plants indicated that they hinder the cell division mechanism thereby leading to a filamentous phenotype, and finally leading to cell death. In addition, the integrity of the bacterial cell membrane is also altered causing cell death. Further gene expression analysis showed that these plant extracts and D-Pinitol hampers with function of Filamenting temperature sensitive-Z (FtsZ) protein which was confirmed through in-vitro inhibition FtsZ –GTPase enzymatic activity.

Keywords : Time kill curve, DPPH, Cell elongation, Bac light assay, GTPase activity, Light scattering, q PCR.

1. Introduction

Mycobacterium tuberculosis is a pathogenic organism which causes Tuberculosis. About one third of the global population is affected with this disease [1]. Due to emerging drug resistant strains, and reduced effectiveness of treatment due to failure in patient adherence to treatment regime leads to complication and failure of treatment [2]. Therefore, there is a need to screen for novel anti-mycobacterial medicinal plant extracts to employ them as complementary and adjuvant medicine along with the conventional chemotherapy to increase the effectiveness and action of chemotherapeutic drugs. Traditionally, plant extracts and their active components have been used to treat many diseases, and the structures of many phytochemicals have been the starting scaffold for the design of synthetic drugs, including aspirin, taxol [3]. Plant extracts possess phenolic compounds and their derivatives play an important role to protect the human body against the damage caused by free radicals [4].

Many plant extracts and compounds were tested against mycobacteria and few were reported for their anti-tubercular activity. Chloroform extracts of *Pterolobium stellatum* (Forssk) (Fabaceae), *Persea americana Mill(Lauraceae)* and *Otostegia integrifolia* Benth (Lamiaceae) have shown MIC of 0.312, 2.5 and 0.312 mg/ml respectively against *M. tuberculosis* strain

H37RV [5]. Methanolic extract of *Aegle marmelos L, Glycyrrhiza glabra L, Lawsonia inermis L, Piper nigrum L* and *Syzygium aromaticum L*, exhibited anti-tuberculosis activity at a range of 0.8 to 100 µg/ml against *M. tuberculosis* strain H37RV [6]. While ethyl acetate extract of *Piper longum* L inhibited *M. smegmatis* at 32 mg/ml [7]. Ethanolic extracts of *Boswellia serrata Roxb.ex*, *Datura stramonium L* and *Lavandula stoechas L* inhibited *M. tuberculosis* strain H37RV with a MIC in the range of 125 to 250 µg/ml [8]. Phytochemicals namely, Distemonanthoside, 4-Methoxygallic acid, Quercetin and Sitosterol 3-*O*-β-D-glucopyranoside inhibited *M. tuberculosis* strain H37RV with a MIC at a range of 31 to 125 µg/ml [9]. Oleanolic acid declined the growth and development of *M. tuberculosis* strain H37RV at a MIC of 50 - 200 µg/ml [10].

FtsZ protein is a bacterial tubulin homolog involved in the creation of a Z-ring at the site of cell division. FtsZ is a Guanosine TriPhosphate (GTP) / Guanosine DiPhosphate (GDP) binding protein with the ability of polymerising GTP-into protofilaments. Abnormalities in polymerization / GTPase activity will lead to the inhibition of Z-ring which makes the cell elongated and finally leads to the death of the organism [11]. This crucial behavior of protein motivated many researchers around the world to focus and design novel inhibitors targeting it. Berberine, chrysophaentins A-H, Cinnamaldehyde, Curcumin and Viriditoxin are potent inhibitors that are known to target GTPase activity of FtsZ [12].

Anti-mycobacterial activity of HXM extracts of three plants namely *Acacia nilotica*, *Aegle* marmelos and *Glycyrrhiza glabra* were studied. *Acacia nilotica, L (A. nilotica)* belongs to the family of Fabaceae commonly known as '*Babul*'. This plant is distributed in all parts of the world. It is used extensively for the treatment of various types of cancers like bone, mouth and skin by traditional healers in different regions of Chattisgarh (India). In West Africa, the root of *A.nilotica* is used to treat tuberculosis, the wood is used to treat smallpox and the leaves are used to treat ulcers [13]. *A. nilotica* extract is used traditionally to treat respiratory related diseases. It has antituberculosis effects and it could serve as lead for developing new antibiotics [14]. Traditionally, the plant roots, leaves, flowers, buds has anticancer, antimicrobial, antioxidant, antimutagenic activity and is used for treating cough, dysentery, leprsosy, opthalmia, small pox, skin ulcers and tuberculosis as well as astringent, antispasmodic and aphrodisiac [15] and [16].

Aegle marmelos (L) Correa *(A.marmelos)* belongs to the Rutaceae family and is also called as bael tree. It is used in indigenous system of Indian medicine. It is native to India and grows wild in Sub-Himalaya from Jhelum, eastwards towards west Bengal, in central and south India [17]. It is known to possess anti-diabetic, anti-cancer, anti-inflammatory and anti-microbial activity [18]. Fruit of *A. marmelos* is also used in the treatment of asthma, dyspepsia, diarrhea, digestive, dysentery, hepatitis, sinusitis, tuberculosis and stomachic etc. [19]. Isolated compounds from fruit extract of *A.marmelos* like coumarins marmelosin, marmin and xanthotoxol and flavonoid kaempferol 3O-rhamnoside, afzelin have been tested against *Mycobacterium tuberculosis* and *Mycobacterium bovis*. Coumarins and marmelosin have shown anti-mycobacterium activity against *M. tuberculosis* H37Ra with an IC_{50} value of 12.46 µg/mL and 4.31 µg/mL [20].

Glycyrrhiza glabra L (*G. glabra*), belongs to the family Fabaceae. It is also called ~~as~~ Licorice. Rhizome and root of *G. glabra* are used as carminative and expectorant by the Chinese, Egyptian, Greek, Indian and Roman civilizations. It is also called ~~as~~ sweet wood and is native to the certain areas of Asia and Mediterranean. It is an old age plant used in traditional medicine for its ethanopharmacological use to cure simple cough to complex cancer and SARS virus [21]. Rhizomes and roots are used orally to treat addison disease, diabetes, lung ailments, kidney stones and tuberculosis. It is also used as mild laxative, contraceptive and to improve sexual function [22]. The roots of *G. glabra* is used traditionally in India to treat pulmonary related diseases, chest ailments, persistent cough and manage tuberculosis [23]. Licorice also shows anti-platelet aggregation effects [24] and it has the ability to relieve cough from ancient times [25].

D-Pinitol, a phytochemical which is predominantly present in all the three HXM extracts (GCMS & HPLC) is studied in detail here. It has the ability to reduce metastasis of human lung cancer [26], it has anti-inflammatory, antihyperlipdemic, cardioprotective and antioxidant activities, [27] and [28]. It is used for cancer of lung, bladder and breast [29] and [30]. It is also effective in the inhibition and progression of prostate cancer [31] and hypoglycemic levels [32].

Initially, anti-mycobacterial screening of Hexane methanolic (HXM) extracts of selected plants were carried out, and the extracts that showed activity were further characterization characterized by Gas Chromatography Mass Spectrophotometer (GCMS) and High Performance

Liquid Chromatography (HPLC). The HXM extracts were tested individually as well as in combination with two current anti-mycobacterial drugs namely Isoniazid and Rifampicin by determining MIC and Synergy. Time-kill studies were done to determine whether the extracts possess bactericidal or bacteriostatic behaviour. Morphology of cells was measured by the cell elongation study. Antioxidant activity of the plant extracts was also studied. The mechanism of action of these HXM extracts / compound were determined by Live/dead assay. The effect of HXM extracts / compound on *M. smegmatis* FtsZ were also investigated by GTPase light scattering and q PCR studies.

2. Materials and Methods

2.1. Bacterial strain

Mycobacterium smegmatis strain (ATCC14468) was procured from the Institute of Microbial Technology, (IMTECH), Chandigarh, India. The bacteria were cultured and maintained at a pH 7, temperature at 37 °C in Middlebrook 7H9 medium, supplemented with OADC (Oleic acid/bovine Albumin, Dextrose, Catalase). These chemicals were procured from Sigma (Aldrich, St. Louis, USA). OADC was prepared separately using, 0.06 ml of oleic acid, 5 g of albumin, 0.85 g of sodium chloride, 2 g of dextrose, 3 mg of catalase and 100 ml of filter sterilized distilled water. It was added separately to the culture medium.

2.2. Plant collection

Leaves of *A. nilotica* were collected from IIT Madras (South India) Campus. Fruits of *A. marmelos* (Bael fruit) were collected from a tree near the campus. The roots of *G. glabra* were purchased from an ayurvedic shop in Chennai (South India). Collected plant samples were identified as leaves of *A. nilotica*, fruits of *A. marmelos* and roots of *G. glabra* by Dr. K.N.Sunil Kumar, Research Officer, HOD Pharmacognosy, Siddha Central Research Institute, Arumbakkam, Chennai, 600106. The samples were deposited in the Herbarium of Siddha Central Research Institute, Arumbakkam, Chennai, 600106**.** The voucher specimens of the above samples are A15061901N, A140619G, G14061901G respectively.

2.3.Herbal preparation

The leaves of *A. nilotica* were thoroughly washed, rinsed with distilled water and dried overnight in a hot air oven at room temperature. Unripened pulp of *A. marmelos* was well macerated and lyophilized into a fine powder. Roots of *G. glabra* were broken into small pieces, washed, rinsed with distilled water and dried overnight in a hot air oven and powdered using a hand blender.

2.4. Extraction procedure

About 20 g of each of the powdered leaves of *A. nilotica,* fruit of *A. marmelos* and roots of *G. glabra* were weighed separately. Each plant powdered sample was defatted with hexane (300 ml) and the mixtures were kept undisturbed overnight with occasional stirring. The hexane treated residues of the above plant parts were filtered separately to remove the fatty volatile mass and partitioned with methanol (150 ml) overnight. Methanol soluble extracts were filtered out, concentrated using a rotavapor in a preweighed flask. This is named as HXM extract.

2.5. Chemicals

Chemicals including Middlebrook 7H9 broth purchased from BD, Biosciences USA, were used in the study to culture *M. smegmatis*. Other chemicals and reagents namely Dimethyl sulfoxide (DMSO), 2,2-diphenyl-1-picrylhydrazyl (DPPH), D-Pinitol, GTP, Isoniazid, Isopropyl β-D-1-thiogalactopyranoside (IPTG) and Rifampicin were procured from Sigma (Aldrich, St. Louis, USA).

2.6. Anti-mycobacterial agents

Anti-mycobacterial activity of the HXM extracts were compared with two standard anti-tubercular drugs namely, Isoniazid (INH) and Rifampicin (RIF). Stock solution of INH (1mg/ml) was prepared with sterile distilled water and RIF (1mg/ml) was prepared with DMSO.

2.7. High performance liquid chromatography (HPLC)

The plant extracts were estimated by HPLC (Shimadzu) at 50°C using a 300 X 7.8 mm^2 column (Phenomenex, Rezex ROA). The eluent used was 5 mM H_2SO_4 at 0.6 ml /min. Extracts of *A. nilotica, A. marmelos* and *G. glabra* were detected by refractive index detector (RID).

Injection volume of the sample was 20 µl. HPLC was followed according to the method as described [33].

2.8. Preparation of D-Pinitol Standard solution

Concentration of D-Pinitol is 1 mg / ml of HPLC grade water. The solution was filtered through 0.45 µ filter paper and 20 µL was injected into the column.

2.9. Gas Chromatography and Mass spectroscopy (GCMS)

The phytochemical investigation of HXM extracts were performed on a GC-MS, Clarus 500 Gas Chromatograph Perkin Elmer, USA. Capillary standard non-polar column measured dimension was 30Mts. Temperature of the oven was set to 50 °C while injection temperature was maintained at 250 °C and injection volume was 1 µl. Plant extracts were rederivatized using BSTFA (*N*,*O*-Bis(trimethylsilyl)trifluoroacetamide).

2.10. Resazurin Assay (REMA)

Resazurin microtitre assay was performed as per the reported protocol [34-36]. To prepare the inoculum, two loops of the bacteria were suspended in 15 ml of Middlebrook 7H9 medium in sterile vial and the culture was kept in orbital shaker at 180 rpm. The cultures were taken for the study once it reached an O.D of 0.6 - 0.7 (JASCO UV Spectrophotomter, 600 nm). Middlebrook 7H9 broth supplemented with OADC (10%) of about 100 µl was added to each well of a 96 well microtitre plate. Plant extract samples were also added in to each well so to arrive at a concentration of 100 mg/ml. Bacterial suspension (100 µl) containing approximately 10^6 CFU/ml was added to all the wells. Controls consisted of cultures with DMSO alone, and anti- tubercular drugs namely Isoniazid and Rifampicin, at 1 mg/ml. The plate was wrapped using aluminium foil and incubated overnight at 37 °C for 48 hour. About 20 µl of resazurin mixed in water (0.2 mg/ml) was added to each well after this incubation period, again wrapped in aluminium foil and incubated for 4 - 5 hours. The change in colour of the solution from blue to pink indicates inhibition while no change indicates the growth of bacteria.

2.11. Synergistic interaction between plant extracts and drugs

Checkerboard broth microdilution method was performed to find the effect of combination of drugs and plant extracts / phytochemical. Briefly, drugs (RIF/INH) are serially

diluted vertically and different concentration of plant extract has been added horizontally in such a way that each concentration of plant extract has been tested in combination with decreasing concentration of the drugs. Middlebrook 7H9 broth of about 100 µl was added into all the wells. Two fold serial dilution of drugs (RIF/INH) has been done vertically and 50 µl aliquots of different concentration of HXM plant extracts / compound were arranged for every conjunction to be tested. 100 µl of grown cultures were added uniformly and incubated for 24 hours at 37 °C. After the incubation, Resazurin dye of about 20 µl (0.02%) was added to all the wells and further incubated for 5 hours. Lowest concentration of antibiotic and plant extracts in combination showing no color change is considered as measure of inhibition of bacterial growth. Synergistic interactions between plant extracts and drugs were calculated by estimating the FIC index, using the formula, (MIC of antibiotic in combination / MIC of antibiotic alone) + (MIC of plant extract in combination / MIC of plant extract alone) [37]. The combination is considered synergistic, additive and antagonist based on the FIC index as < 0.5, 1 or > 1.5 respectively.

2.12. Time Kill assay

Combinations of drugs with extracts were employed equivalent to 1 x MIC. 10 ml of treatment mixtures were arranged and inoculated with 20 µl of 2-day old culture to reach a final Optical Density (O.D) of 0.6 (i.e. $2x10^7$ cells). 50 µl aliquots were withdrawn at different time periods starting from (0, 1, 2, 3, 4, 5, 6 and 7 days) and diluted serially and corresponding dilution was spread plated on 7H10 agar and incubated at 37°C. Bacterial cell colonies were enumerated by help of magnifying lens, and a kill curve response has been plotted between time and Log_{10} Colony Forming Unit (CFU) to determine the mode of action of the combination [38].

2.13. Bacteria Live / Dead assay

Live / Dead assay was performed as per manufacturers protocol using Baclight kit method [39]. Briefly, the bacterial cultures were grown at 37°C and centrifugation done at 10,000 rpm for 10 minutes at 4 °C and the supernatant collected were discarded. They were washed in sterile water. HXM extracts, D-Pinitol, INH/ RIF were added at 1 x MIC and this setup was kept at 37 °C for 4 hr. After incubation, equal amount of Bac light reagent was added, the vials were covered in aluminum foil and incubated at 37 °C for 15 minutes. After this, around 2- 5 µl of above samples were mounted on the microslide for viewing the dead and live cells using 40 x objective using a Carl-Zeiss Fluorescence Microscope (Axio Imager 2 for Life

Science Research, Germany). The kit consists of SYTO9 (green fluorescence dye) is read at 480/500 nm and propidium iodide (red fluorescence dye) is read at 490/635 nm**.** The former stains the bacteria with both intact as well as damaged membranes, and the latter stains only the cells with damaged membranes.

2.14. Antioxidant activity (DPPH assay)

Antioxidant activity of the plant extracts were assessed based on the radical scavenging activity of DPPH [40]. HXM extracts / D-Pinitol of 25 µl (100 µg/ml and 0.50 µM solution respectively) were added to freshly prepared DPPH solution (1ml) into test tubes separately. Absorption was measured at 515 nm after 20 minutes of incubation. Extracts without DPPH were used as the blank and DPPH in methanol served as the control. Percentage of the radical scavenging activity of the samples were calculated as (OD control - OD sample) / OD control) × 100.

2.15. Cell elongation studies

The effect of above plant extracts and D-Pinitol on the increase in the *M. smegmatis* cells was determined. Briefly, overnight *M. smegmatis* grown culture (0.6-0.8 O.D) were treated separately with plant extracts and D-Pinitol (1 x MIC) and incubated for 4 hours at 37 °C. Centrifugation was done for treated samples at 10,000 rpm for 10 minutes at 4 °C. Supernatant collected was removed and the pellets were washed in sterile Phosphate buffered saline (PBS). This process is repeated two times. About 0.1 - 0.2 µl of the washed cell pellets were smeared on a clean microscopy slide. Cell morphology was observed under a Carl-Zeiss Bright Field Microscopy (Axio Imager 2 for Life Science Research, Germany) and the elongated cell length was measured using Image J software.

2.16. Inhibition of GTPase:

pSAR1 plasmid harbouring *M. tuberculosis* FtsZ gene construct in pET15 vector was a generous gift from Dr.Malini Rajagoplan, University of Texas, USA. Plasmid was expressed in BL21DE3 E.coli strain exposed to IPTG (1mM) and purified by Ni-NTA column chromatography. Expression and purification of the protein was carried according to method described [41] and the purity was checked by SDS-PAGE analysis. Inhibition of GTPase was studied according to the method described [12]. Briefly, hydrolysis reaction was carried out using FtsZ (12 µM) and GTP (1 mM) in 50 µl reaction buffer with / without HXM extracts and

compounds. They are incubated for 30 minutes at 37 °C. Post 30 minutes 0.5 mM Ethylenediaminetetraacetic acid (EDTA) was added to quench the reactions and aliquots of about 5 µl was drawn and 120 µl of colour reagent was added and incubated again for 5 minutes at room temperature. This was followed by the addition of 30 µl of 34% Sodium Citrate. Absorbance was read against 630 nm using Enspire plate reader (Perkin-Elmer LS-55, Sparta, NJ). A standard graph also prepared with pure KH_2PO_4.

2.17. FtsZ gene expression study

To ascertain the effect of the HXM extracts / compound on the gene expression of FtsZ, real time-PCR was done as explained by [42] with some variations. The housekeeping gene, 16srRNA rrsB of *Mycobacterium* was used as reference and using this FtsZ gene fold expression was computed by $2^{-(\Delta\Delta Ct)}$ model [43]. *M. smegmatis* culture at O.D of 0.1 was treated with HXM extracts / compound at 1.5 X MIC and RNA isolation was done by Trizol Reagent method [44]. Isolated messenger Ribonucleic acid (mRNA) was transformed to its corresponding complementary deoxyribonucleic acid (cDNA) using cDNA synthesis kit (Takara Bio Inc) and quantified with qPCR, by employing SYBR green reporter. Molecular Probe to obtain the cycle threshold-C_t values of untreated (normal) and treated cells. Primers used in the study of FtsZ gene expression are listed in the supplementary file.

2.18. Statistical Analysis

All the experiments were conducted in triplicates and values are depicted as Mean ± Standard Deviation. One-way ANOVA has been performed on required cases to verify the statistical significance between the treatments.

3. Results and Discussion

There has been no new anti tuberculosis drug introduced in the past 30 years. Also, due to the resistance acquired by the strain of already existing drug, development of novel and effective drug inhibiting newer targets becomes an urgent need [45]. Medicinal plants and their constituents could possibly help in identifying new drugs to overcome the disease.

3.1. Characterisation of Plant extracts by GCMS and HPLC

3.1.1. Gas Chromatography Mass Spectrometry (GCMS) analysis

The various active compounds as identified by the database with their peak number, concentration (peak area %), and retention time (RT) of all three plant extracts and their biological activities of the phytochemicals identified by GCMS analysis are provided in the supplementary material.

3.1.2 High performance liquid chromatography (HPLC)

The compound D-Pinitol is kept as a standard to internalize the comparison with all the three extracts. Standard D-Pinitol produced the optimized peak at 10.198 retention time at 10th minute. Similarly, extracts of *A. nilotica, A. marmelos* and *G. glabra* showed the presence of D-Pinitol at the retention time of 10.187, 10.143 and 10.135 respectively at the 10th minute. This statement, clearly pinpoints the presence of natural compound D-Pinitol in the above three HXM extracts. So, D-Pinitol is considered as a compound of interest and taken for further study.

D-Pinitol commonly called as, 3-O-methyl D-Chiro inositol, is reported to have pharmacological significance, including anti-diabetic, anti-inflammatory, antioxidant and immunosuppressive potential [46]. It is used to treat AIDS, cardiovascular problems, hypertension, rheumatism and certain neurological problems [47]**.** D-Pinitol, a well known anti-diabetic agent which acts by reducing the hyperglycemic levels [48].

3.2. Anti-mycobacterial activity of plant extracts

The anti-mycobacterial activity of the HXM extracts of *A. nilotica, A. marmelos* and *G. glabra* are 1.56 ± 0.03 mg/ml, 1.32 ± 0.02 mg/ml, 1.25 ± 0.03 mg/ml, respectively indicating that hexane wash of the plants enhances the activity. D-Pinitol, a predominant phytochemical found in these plants (based on GCMS and HPLC profile) showed MIC of 0.58 ± 0.01 µM, while commercial drugs namely Isoniazid and Rifampicin had a MIC of 29 ± 0.06 µM and 2.43 ± 0.04 µM respectively.

Others have also reported that the activity of different plant extracts depend on the solvent used for extraction. Hexane extract of *A. marmelos* fruit inhibited *M. tuberculosis* strain H37RV growth at 50 µg/ml while methanol extract inhibited at 100 µg/ml [49]. Acetone extract of *G. glabra* root inhibited *M. tuberculosis* strain H37RV in the range of 0.97 - 1.95 µg/ml [3]. Methanol followed by ethylacetate fraction of pomegranate peel inhibited *M. smegmatis* mc^2155 between 3.35 mg/ml to 0.395 mg/ml [12]. Ethylacetate extract of *Piper longum*

inhibited *M. smegmatis* at 64.09 mg/ml [7]. Glycyrrhizin, a phytochemical from *G. glabra* inhibited *M. tuberculosis* strain H37RV at 100 μg/ml [50]. The plants in this study seem to exhibit better activity when compared to research in the literature.

3.3. Synergistic interaction between plant extracts / D-Pinitol and commercial drugs like Isoniazid and Rifampicin

The extracts of *A. nilotica, A. marmelos, G. glabra* and D-Pinitol are studied in conjunction with Isoniazid and Rifampicin [51][51](Khadkikar et al., 2018)in different proportions to test the effect of combination therapy against *M. smegmatis*. The results indicated that, these at sub-minimal inhibitory concentration were able to reduce the MIC of both the drugs. HXM extract of *A. nilotica* (1.56 mg/ml) reduced the MIC of INH from 29 μM to 0.14 μM and MIC of RIF from 2.43 μM to 0.01 μM (FIC index = 0.33 and 0.24 respectively). HXM extract of *A. marmelos* (1.32 mg/ml) reduced the MIC of INH from 29 μM to 0.43 μM and that of RIF from 2.43 μM to 0.01 μM (FIC index = 0.24 and 0.26 respectively). HXM extract of *G. glabra* (1.25 mg/ml) decreased the MIC of INH from 29 μM to 0.21 μM and that of RIF from 2.43 μM to 0.01 μM (FIC index = 0.17 and 0.15 respectively). D-Pinitol (0.58 μM) reduced the MIC of INH from 29 μM to 0.07 μM and that of RIF from 2.43 μM to 0.03 μM (FIC index = 0.21 and 0.19) respectively. These results indicate that these three HXM plant extracts and D-Pinitol combine in synergistic fashion with these two drugs their by reducing their concentration by (8-10 folds) required to inhibit *M.smegmatis*.

Published literature indicated that, MIC of RIF is reduced by 64-fold in combination with the acetone extract of *Cremaspora triflora* Thonn against *M. smegmatis* ATCC 1441 [52]. MIC of Isoniazid is reduced by 8 fold in conjunction with ethyl acetate extract of *Knowltonia vesicatoria L* against *M. smegmatis* mc2 155 [53]. Plumbagin, from *Plumbago zeylanica L* and Ferelenol from *Ferelenol communis* reduced the MIC of Isoniazid by 8 fold against *M.tuberculosis* strain H37RV [54]. Also, Curcumin and demethoxycurcumin reduced the MIC of Isoniazid by 16 fold against *M. smegmatis* mc^2 155 [55]. MIC of Rifampicin reduced from 0.015 to 0.00048 mg/ml when given in combination with antitubercular drug SQ109 against *M. tuberculosis* strain H37RV [56]. Cumene hydroperoxide reduced the MIC of Rifampicin by 16-fold against *M. tuberculosis* strain H37RV [57].

Though D-Pinitol has been considered as the lead component to test for activity since being a major fraction in GCMS and HPLC analysis, it can also be noted that in addition to D-Pinitol other phytochemicals present in the HXM extracts may also contribute to the anti-mycobacterial activity which may require separate study. The results of the combination study shown in the form of isobologram (Figure 1 and Figure 2) suggests that HXM extracts could be used in supplementation along with the drugs and to decrease the concentration of the latter for managing tuberculosis. This could result in reducing the toxicity of the drug as well as addressing the chance of emergence of resistance strains.

3.4. Time–kill studies

Extracts of *A. nilotica, A. marmelos* and *G. glabra* and D-Pinitol were employed for time-kill studies individually and in combinations with INH and RIF. An average of 3 Log fold decline in the growth rate of *M. smegmatis* post 48 hours when compared to the untreated control was observed in all the cases. Also the extracts of these plants in combination with INH and RIF showed an average 2 Log reduction in viable cell count when compared to untreated while other *A.nilotica* extract in combination showed an equivalent kill-profile similar to INH and RIF alone. To confirm whether the growth rate retardation activity is governed by time or due to the presence of active constituents of individual extracts, the activity of D-Pinitol in combination, was also investigated which also showed that in combination with INH (Figure 3a.) and RIF (Figure 3b) a 2 Log retardation in the growth of viable cell count when compared to control was observed. Literature reports that, dihydrofusarubin and Rifampicin combination showed bacteriostatic activity [58]. Ethanolic extract of *Andrographis paniculata* Burm showed bacteriostatic activity against *M. tuberculosis* in combination with Isoniazid [59].

3.5. Live/ Dead assay

Membrane damage of *M. smegmatis* cell wall after incubating with HXM extracts and D-Pinitol were determined using a Fluorescent Microscope, after staining with STY09 and PI (Figure 4a-4g.). Green indicates live and red indicates dead cells, since PI penetrates the damaged cell wall. Treatment with *A. marmelos, A. nilotica, G. glabra* and D-Pinitol leads to 43.4%, 55.1%, 49.1% and 50.4% cell wall damage respectively (Figure 4h.). Percentage of cell death on treatment with INH and RIF are 31.7% and 49.3% respectively.

Membrane structural integrity of bacterial cell plays a vital factor for the function of the cell membrane by internally maintaining cellular conditions required for metabolism [60].Numerous studies have reported that, plant phytoconstituents play a role in rupturing cell membrane by affecting its structural integrity. Curcumin I was reported to be active against both Gram positive and negative bacteria, with 100% killing at a dose of 100 μM [61]**.** Present study indicates that if the concentration of D-Pinitol is increased from 3 μM to 10 μM, probably, 100% cell membrane damage could be achieved which suggests its efficacy at lower concentration than other compounds reported in literature.

3.6 Antioxidant activity (DPPH assay)

Extracts of *A. nilotica, A. marmelos, G. glabra* and D-Pinitol exhibited DPPH radical scavenging activity of 86.2 ± 2.2%, 72.6 ± 1.2%, 79.9 ± 0.7% and 69.4 ± 1.8% respectively indicating high antioxidant activity. So they may also act by inhibiting the enzymes responsible for free radical production, by scavenging the reactive oxygen species or by shielding the anti-oxidant defense system.

The current results are similar to reported literature which states that many plant species including *A. biebersteinii, A. austriaca, A. tinctoria var. tinctoria, A. pseudocotula, C. oblonga, P. major* (leaf), *P. spina-christi, R. sanctus* (shoot, flower, leaf), *R. canina* (leaf, shoot, flower, root*), T. polium, S. verticillata,* and *S. fruticosa* exhibit anti-oxidant activities in the range from 81.4 ± 0.2% to 86.8 ± 1.4% [62].

3.6. Cell elongation study

Microscopic images of *M. smegmatis* on exposure to the plant extracts, the phytochemical D-Pinitol and drugs are shown in Figure 5a-5g. The mean length of *M. smegmatis* (3.9 ± 0.5 µm) on treatment with HXM extract of *A. nilotica* (8.2 ± 1.0 µm), *A. marmelos* (15.7 ± 1.2 µm), *G. glabra* (13.5 ± 0.5 µm) and D-Pinitol (12.7 ± 0.1 µm) increased by three to five times (Figure 5h.). INH and RIF treated *M. smegmatis* cells also exhibited elongation (9.7 ± 0.6 µm and 11.5 ± 0.2 µm).

Plumbagin and totarol increased the bacteria length by seven and five folds [63]. Length of *M. smegmatis* bacteria increased to three to four folds on treatment with Esculetin and

Scopuletin (100 μM) [12]. We also observed similar results with these HXM extracts and D-Pinitol indicating that they probably act by inhibiting the first stage of cell division proteins, namely FtsE, FtsX and FtsZ by inducing cell elongation thereby inducing cell death. The elongated cells could not undergo cell division this could be due to the nonfunctional septum formation by their action.

3.7. Effect of plant extracts on *M. smegmatis* FtsZ GTPase activity

Since the plant extracts were shown to interfere with cellular morphology leading to an elongated cells. The effect of these plant extracts on cell division were probed. Since FtsZ is the prime protein that acts as a choreographer to organize and assemble other cell division machinery, we probed the effect of HXM extracts of these plants over FtsZ activity.

A. nilotica, *A. marmelos* and *G. glabra* inhibited the *M.tb* FtsZ GTPase activity with an IC_{50} of 22.50 ± 1.25, 23.45 ± 1.12 and 37.9 ± 1.19 mg/ml respectively (Figure 6 a & b). D-Pinitol inhibited GTPase activity with an IC_{50} of 18.71 ±0.9 μM. Berberine, served as a positive control here and it inhibited the activity of GTPase by 19.41 ± 1.1 μM respectively. Compounds like 7-dimethyl-4- methylcoumarin, daphnetin and esculetin have also been reported to inhibit this activity [12]. GTPase activity of FtsZ protein is essential for the creation of Z-ring and abnormalities in this function caused by plant based inhibitors will lead to cell death.

3.8. Polymerisation of FtsZ

In order to further confirm the effect of extracts on polymerisation of the FtsZ, a light scattering experiment is performed using HXM extracts / D-Pinitol at 1.5 X MIC. All the three plant extracts showed a decrease in the rate of protofilament by 50% after 300 seconds, whereas D-Pinitol showed a stable reduction (Figure 6c). Many natural compounds from plant extracts such as Cinnamic acid, Curcumin [64] and Berberine [65] are reported to inhibit the polymerisation activity.

3.9. Studies on gene expression of FtsZ

The FtsZ gene expression increased by 2.15, 3.34 and 4.19 fold on treatment with *A. nilotica*, *A. marmelos* and *G. glabra* when compared to untreated. D-Pinitol treatment increased the FtsZ gene expression by about 2.80 fold (Figure 6d.). This indicates that these HXM extracts

/ D-Pinitol alters the gene expression of FtsZ considerably in addition to affecting the activity of the FtsZ protein. Many natural phenolic compounds and synthetic derivatives of coumarins are reported for the increased expression of *M. tuberculosis* FtsZ [51].

4. Conclusions

M. smegmatis, a non pathogenic bacteria, which is closer to *M. tuberculosis* in sequence similarity is used in our study to find the effect of plant extracts against it. The invitro synergistic activity of extracts of *A. nilotica*, *A. marmelos* and *G. glabr*a and the phytochemical D-Pinitol (which is found in all three plants extracts) in combination with two drugs namely Isoniazid and Rifampicin were also studied. Plant extracts are known to act on multiple targets (Figure 7.) while Isoniazid is known to inhibit the mycolic acid production, a cell wall component and Rifampicin is known to act by inhibiting the bacterial DNA dependent RNA synthesis by inhibiting the RNA.

HXM extracts and the phytochemical, D-Pinitol produced synergistic effects at different subminimal concentration when combined with the drugs. In time kill analysis, treatment of log-phase *M. smegmatis* with 1 x MIC concentration resulted in 2 to 3 Log reduction in viable cells indicating that HXM extracts exhibit bacteriostatic activity. HXM extracts and D-Pinitol also induce damage to the cell membrane of *M. smegmatis* by affecting its structural integrity thereby causing cell death.

D-Pinitol is an interesting biological niche that acts as a free radical scavenger and it inhibits *M. smegmatis* growth, probably by perturbing the cell division process which resulted in an elongated morphology. This implies that it could be a potential lead as an anti- mycobacterial agent in drug development. So, for the first time the current study has identified a new phytochemical, which could lead a way towards the development of novel anti- mycobacterial drug. Also, synergy study has indicated that concentration of commercial drug such as Isoniazid and Rifampicin could be reduced considerably by combining them with HXM plant extracts which could lead to reduced toxicity of the former and address the emergence of resistant strains.

The current study indicates that the chosen plant extracts / D-Pinitol can be explored for potential leads in tuberculosis therapy. The study has also identified that they affect the cell division process by inhibiting the GTPase activity of Mycobacterium-FtsZ. These plant extracts

also altered the expression of *Mycobacterium*-FtsZ gene. Of course, the pharmacokinetics and pharmcodynamics of these combinations need to be studied in animal models before these could be actually taken up further as adjuvant therapy to potentiate the action of commercial drugs or as complementary and alternative therapy.

Conflict of Interest

We have no conflict of interest.

Acknowledgement

We also thank Prof. Anju Chada and Prof. Guhan Jayaraman, Department of Biotechnology, IIT Madras for analyzing our plant extracts for GCMS and HPLC.

References

1. Bishai JD, Bishai WR, Bishai DM (2010) Heightened vulnerability to MDR-TB epidemics after controlling drug-susceptible TB. PLoS One 5: e12843.
2. Andrews JR, Sarita Shah N, Gandhi N, Moll T, Friedland G (2007) Multidrug-resistant and extensively drug-resistant tuberculosis: implications for the HIV epidemic and antiretroviral therapy rollout in South Africa. The Journal of infectious diseases 196: S482-S490.
3. Nair SS, Pharande RR, Bannalikar AS, Mukne AP (2015) In vitro antimycobacterial activity of acetone extract of Glycyrrhiza glabra. Journal of Pharmacy & Pharmacognosy Research 3: 80-86.
4. Gowrish A, Vagdevi H, Rajashekar H (2015) In vitro antioxidant and antitubercular activity of Leucas marrubioides Desf. root extracts. Journal of Applied Pharmaceutical Science 5: 137-142.
5. Kahaliw W, Aseffa A, Abebe M, Teferi M, Engidawork E (2017) Evaluation of the antimycobacterial activity of crude extracts and solvent fractions of selected Ethiopian medicinal plants. BMC complementary and alternative medicine 17: 143.
6. Kaur R, Kaur H (2015) Antitubercular activity and phytochemical screening of selected medicinal plants. Orient J Chem 31: 597-600.
7. Barua C, Singh A, Sen S, Barua A, Barua I (2014) In vitro antioxidant and antimycobacterial activity of seeds of Piper longum Linn: a comparative study. SAJ Pharm Pharmacol 1: 2375-2262.2371.
8. Rahgozar N, Bakhshi Khaniki G, Sardari S (2018) Evaluation of Antimycobacterial and Synergistic Activity of Plants Selected Based on Cheminformatic Parameters. Iranian biomedical journal: 0-0.
9. Evina JN, Bikobo DSN, Zintchem AAA, Nyemeck I, Mbabi N, et al. (2017) In vitro antitubercular activity of extract and constituents from the stem bark of Disthemonanthus benthamianus. Revista Brasileira de Farmacognosia 27: 739-743.
10. Ge F, Zeng F, Liu S, Guo N, Ye H, et al. (2010) In vitro synergistic interactions of oleanolic acid in combination with isoniazid, rifampicin or ethambutol against Mycobacterium tuberculosis. Journal of medical microbiology 59: 567-572.
11. Bernander R, Ettema TJ (2010) FtsZ-less cell division in archaea and bacteria. Current opinion in microbiology 13: 747-752.

12. Sridevi D, Sudhakar KU, Ananthathatmula R, Nankar RP, Doble M (2017) Mutation at G103 of MtbFtsZ Altered their Sensitivity to Coumarins. Frontiers in microbiology 8: 578.
13. Kalaivani T, Mathew L (2010) Free radical scavenging activity from leaves of Acacia nilotica (L.) Wild. ex Delile, an Indian medicinal tree. Food and Chemical Toxicology 48: 298-305.
14. Oladosu P, Isu N, Ibrahim K, Orishadipe A, Lawson L (2013) Antituberculosis activity of bioactiv e compounds from fruit extract of Acacia nilotica. J Microbiol Res 3: 247-254.
15. Farzana M, Tharique I (2014) A review of ethnomedicine, phytochemical and pharmacological activities of Acacia nilotica (Linn) willd. Journal of Pharmacognosy and Phytochemistry 3: 84-90.
16. Ali A, Akhtar N, Khan BA, Khan MS, Rasul A, et al. (2012) Acacia nilotica: a plant of multipurpose medicinal uses. Journal of medicinal plants research 6: 1492-1496.
17. Lambole V, Murti K, Kumar U, Bhatt S, Gajera V (2010) Phytopharmacological properties of Aegle marmelos as a potential medicinal tree: an overview. Int J Pharm Sci Rev Res 5: 67-72.
18. Rahman S, Parvin R (2014) Therapeutic potential of Aegle marmelos (L.)-An overview. Asian Pacific journal of tropical disease 4: 71.
19. Solanki R, Mathur V, Purohit SK, Mathur M (2017) Evaluation of wound healing activity of ethanolic extract of Ocimum basilicum and Aegle marmelos leaves in male albino rats. International Journal of Drug Research and Technology 2: 4.
20. Chinchansure A, Shamnani N, Arkile M, Sarkar D, Joshi S (2015) Antimycobacterium activity of coumarins from fruit Pulp of Aegle marmelos (l.) Correa. International Journal of Basic and Applied Chemical Sciences 5: 39-44.
21. Saxena S (2005) Glycyrrhiza glabra: medicine over the millennium.
22. Al-Snafi AE (2018) Glycyrrhiza glabra: A phytochemical and pharmacological review. IOSR Journal of Pharmacy 8: 1-17.
23. Gupta VK, Kaushik A, Chauhan DS, Ahirwar RK, Sharma S, et al. (2018) Anti-mycobacterial activity of some medicinal plants used traditionally by tribes from Madhya Pradesh, India for treating tuberculosis related symptoms. Journal of ethnopharmacology 227: 113-120.
24. Yu Z, Ohtaki Y, Kai K, Sasano T, Shimauchi H, et al. (2005) Critical roles of platelets in lipopolysaccharide-induced lethality: effects of glycyrrhizin and possible strategy for acute respiratory distress syndrome. International immunopharmacology 5: 571-580.
25. Sedighinia F, Afshar AS (2012) Antibacterial activity of Glycyrrhiza glabra against oral pathogens: an in vitro study. Avicenna journal of phytomedicine 2: 118.
26. Sethi G, Ahn KS, Sung B, Aggarwal BB (2008) Pinitol targets nuclear factor-κB activation pathway leading to inhibition of gene products associated with proliferation, apoptosis, invasion, and angiogenesis. Molecular Cancer Therapeutics 7: 1604-1614.
27. NUMATA A, TAKAHASHI C, FUJIKI R, KITANO E, KITAJIMA A, et al. (1990) Plant Constituents Biologically Active to Insects. VI.: Antifeedants for Larvae of the Yellow Butterfly, Eurema hecabe mandarina, in Osmunda japonica.(2). Chemical and pharmaceutical bulletin 38: 2862-2865.
28. Geethan PA, Prince PSM (2008) Antihyperlipidemic effect of D-pinitol on streptozotocin-induced diabetic wistar rats. Journal of biochemical and molecular toxicology 22: 220-224.
29. Zhan T, Lou H (2007) Synthesis of azole nucleoside analogues of D-pinitol as potential antitumor agents. Carbohydrate research 342: 865-869.
30. Rengarajan T, Nandakumar N, Balasubramanian MP (2012) D-Pinitol attenuates 7, 12 dimethylbenz [a] anthracene induced hazards through modulating protein bound carbohydrates, adenosine triphosphatases and lysosomal enzymes during experimental mammary carcinogenesis. Journal of experimental therapeutics & oncology 10.
31. Lin T-H, Tan T-W, Tsai T-H, Chen C-C, Hsieh T-F, et al. (2013) D-pinitol inhibits prostate cancer metastasis through inhibition of αVβ3 integrin by modulating FAK, c-Src and NF-κB pathways. International journal of molecular sciences 14: 9790-9802.

32. Gao Y, Zhang M, Wu T, Xu M, Cai H, et al. (2015) Effects of D-pinitol on insulin resistance through the PI3K/Akt signaling pathway in type 2 diabetes mellitus rats. Journal of agricultural and food chemistry 63: 6019-6026.
33. Kaur M, Jayaraman G (2016) Hyaluronan production and molecular weight is enhanced in pathway-engineered strains of lactate dehydrogenase-deficient Lactococcus lactis. Metabolic engineering communications 3: 15-23.
34. Banfi E, Scialino G, Monti-Bragadin C (2003) Development of a microdilution method to evaluate Mycobacterium tuberculosis drug susceptibility. Journal of antimicrobial chemotherapy 52: 796-800.
35. Palomino J-C, Martin A, Camacho M, Guerra H, Swings J, et al. (2002) Resazurin microtiter assay plate: simple and inexpensive method for detection of drug resistance in Mycobacterium tuberculosis. Antimicrobial agents and chemotherapy 46: 2720-2722.
36. Taneja NK, Tyagi JS (2007) Resazurin reduction assays for screening of anti-tubercular compounds against dormant and actively growing Mycobacterium tuberculosis, Mycobacterium bovis BCG and Mycobacterium smegmatis. Journal of antimicrobial chemotherapy 60: 288-293.
37. Bapela N, Lall N, Fourie P, Franzblau S, Van Rensburg C (2006) Activity of 7-methyljuglone in combination with antituberculous drugs against Mycobacterium tuberculosis. Phytomedicine 13: 630-635.
38. Hemaiswarya S, Doble M (2010) Synergistic interaction of phenylpropanoids with antibiotics against bacteria. Journal of Medical Microbiology 59: 1469-1476.
39. Lahiri R, Randhawa B, Krahenbuhl J (2005) Application of a viability-staining method for Mycobacterium leprae derived from the athymic (nu/nu) mouse foot pad. Journal of medical microbiology 54: 235-242.
40. Suryanarayanan TS, Venkatachalam A, Thirunavukkarasu N, Ravishankar JP, Doble M, et al. (2010) Internal mycobiota of marine macroalgae from the Tamilnadu coast: distribution, diversity and biotechnological potential. Botanica Marina 53: 457-468.
41. Chen Y, Anderson DE, Rajagopalan M, Erickson HP (2007) Assembly dynamics of Mycobacterium tuberculosis FtsZ. Journal of Biological Chemistry 282: 27736-27743.
42. Li Z, Garner AL, Gloeckner C, Janda KD, Carlow CK (2011) Targeting the Wolbachia cell division protein FtsZ as a new approach for antifilarial therapy. PLoS neglected tropical diseases 5: e1411.
43. Livak KJ, Schmittgen TD (2001) Analysis of relative gene expression data using real-time quantitative PCR and the 2− ΔΔCT method. methods 25: 402-408.
44. Rio DC, Ares M, Hannon GJ, Nilsen TW (2010) Purification of RNA using TRIzol (TRI reagent). Cold Spring Harbor Protocols 2010: pdb. prot5439.
45. Amir M, Khan MA, Ahmad S, Akhtar M, Mujeeb M, et al. (2016) Ameliorating effects of Tamarindus indica fruit extract on anti-tubercular drugs induced liver toxicity in rats. Natural product research 30: 715-719.
46. Chauhan PS, Gupta KK, Bani S (2011) The immunosuppressive effects of Agyrolobium roseum and pinitol in experimental animals. International immunopharmacology 11: 286-291.
47. Poongothai G, Sripathi SK (2013) A review on insulinomimetic pinitol from plants. Int J Pharm Bio Sci 4: 992-1009.
48. Misra L, Siddiqi S (2004) Dhaincha (Sesbania bispinosa) leaves: A good source of antidiabetic (+)-pinitol. Current science 87: 1507-1507.
49. Suja K, Jose L, Lakshmanan D, Vidya AG, Reshmi Nair R, et al. (2017) Isolation and Characterization of Antimycobacterial Compounds from Fruits of Aegle marmelos (L.) Correa. J Commun Dis 49: 4.

50. Chidambaram S, Swaminathan R (2016) IN-VITRO ANTIMYCOBACTERIAL ACTIVITY OF SELECTED INDIAN MEDICINAL PLANTS TO RESISTANT STRAINS OF MYCOBACTERIUM TUBERCULOSIS. International Journal of Pharmaceutical Sciences and Research 7: 4130.

51. Khadkikar P, Goud NS, Mohammed A, Ramamoorthy G, Ananthathatmula R, et al. (2018) An efficient and facile green synthesis of bisindole methanes as potential Mtb FtsZ inhibitors. Chemical biology & drug design 92: 1933-1939.

52. Aro AO, Dzoyem JP, Eloff JN, McGaw LJ (2016) Extracts of six Rubiaceae species combined with rifampicin have good in vitro synergistic antimycobacterial activity and good anti-inflammatory and antioxidant activities. BMC complementary and alternative medicine 16: 385.

53. Labuschagné A, Hussein AA, Rodríguez B, Lall N (2012) Synergistic antimycobacterial actions of Knowltonia vesicatoria (Lf) Sims. Evidence-based complementary and alternative medicine 2012: 9.

54. Mossa JS, El-Feraly FS, Muhammad I (2004) Antimycobacterial constituents from Juniperus procera, Ferula communis and Plumbago zeylanica and their in vitro synergistic activity with isonicotinic acid hydrazide. Phytotherapy Research: An International Journal Devoted to Pharmacological and Toxicological Evaluation of Natural Product Derivatives 18: 934-937.

55. Lechner D, Gibbons S, Bucar F (2008) Modulation of isoniazid susceptibility by flavonoids in Mycobacterium. Phytochemistry Letters 1: 71-75.

56. Reddy VM, Einck L, Andries K, Nacy CA (2010) In vitro interactions between new antitubercular drug candidates SQ109 and TMC207. Antimicrobial agents and chemotherapy 54: 2840-2846.

57. Patel YS, Mehra S (2017) Synergistic Response of Rifampicin with Hydroperoxides on Mycobacterium: A Mechanistic Study. Frontiers in microbiology 8: 2075.

58. Bao Z, Cui Y, Sheng Y, Zhu M (2017) Dihydrofusarubin and rifampicin combination prevents pulmonary tuberculosis in murine models of tuberculosis. Int J Clin Exp Med 10: 12343-12348.

59. Bhatter P, Gupta P, Daswani P, Tetali P, Birdi T (2015) Antimycobacterial Efficacy of Andrographis paniculata Leaf Extracts Under Intracellular and Hypoxic Conditions. Journal of Evidence-Based Complementary & Alternative Medicine 20: 3-8.

60. Sánchez E, García S, Heredia N (2010) Extracts of edible and medicinal plants damage membranes of Vibrio cholerae. Applied and Environmental Microbiology 76: 6888-6894.

61. Tyagi P, Singh M, Kumari H, Kumari A, Mukhopadhyay K (2015) Bactericidal Activity of Curcumin I Is Associated with Damaging of Bacterial Membrane. PLOS ONE 10: e0121313.

62. Orhan DD, ÖZÇELİK B, HOŞBAŞ S, Vural M (2012) Assessment of antioxidant, antibacterial, antimycobacterial, and antifungal activities of some plants used as folk remedies in Turkey against dermatophytes and yeast-like fungi. Turkish Journal of Biology 36: 672-686.

63. Bhattacharya A, Jindal B, Singh P, Datta A, Panda D (2013) Plumbagin inhibits cytokinesis in B acillus subtilis by inhibiting F ts Z assembly–a mechanistic study of its antibacterial activity. The FEBS journal 280: 4585-4599.

64. Rastogi N, Domadia P, Shetty S, Dasgupta D (2008) Screening of natural phenolic compounds for potential to inhibit bacterial cell division protein FtsZ. Indian J Exp Biol 46: 783-787.

65. Domadia PN, Bhunia A, Sivaraman J, Swarup S, Dasgupta D (2008) Berberine targets assembly of Escherichia coli cell division protein FtsZ. Biochemistry 47: 3225-3234.

Figure Legends

Figure 1. Interaction of Isoniazid with HXM extracts of a) *A. nilotica,* b) *A. marmelos* and c) *G. glabra* and d) the phytochemical, D-Pinitol against *M. smegmatis*. Line indicates additivity. Point below the line indicates synergistic interaction between the two drugs. FIC Index a) 0.33, b) 0.24, c) 0.17, d) 0.21.

Figure 2. Interaction of Rifampicin with extracts of a) *A. nilotica*, b) *A. marmelos* and c) *G. glabra*, and d) the phytochemical, D-Pinitol against *M. smegmatis.* Line indicates additivity. Points below the line indicates synergistic interaction between the two. FIC Index a) 0.24, b) 0.26, c) 0.15, d) 0.19.

Figure 3. Time Kill curves of a) Isoniazid, b) Rifampicin in combination with the phytochemical D-Pinitol against *M. smegmatis*

Figure 4. Fluoroscence images of *M. smegmatis* cells on treatment with a) Control, b) Isoniazid, c) Rifampicin, d) *A. marmelos*, e) *A. nilotica*, f) *G. glabra* and g) D-Pinitol [Green indicate viable and Red indicates dead (membrane damaged) cells] h) % of Dead cells on treatment (estimated using Baclight ® dual staining kit).

Figure 5. Elongation of *M. smegmatis* cells on treatment with a) Control, b) Isoniazid, c) Rifampicin, d) *A. marmelos,* e) *A. nilotica*, f) *G. glabra* and g) D-Pinitol, h) Average length of *M. smegmatis* cells on treatment. Arrow indicates elongated cells.

Figure 6. Effect of a) HXM extracts (mg/ml) and b) D-Pinitol (µM) on *M. smegmatis* FtsZ - GTPase polymerisation activity. c) FtsZ polymerisation activity measured using 90° light scattering assay at 1.5 X MIC with FtsZ at 12 µM. Each point represents the mean of three different replicates, d) RT – PCR Gene expression analysis - fold increase in the expression of FtsZ post treatment at 1.5 X MIC.

Figure 7. Mechanism of action of HXM extracts, D-Pinitol and commercial drugs on *M. smegmatis* cells. HXM plant extracts and D-Pinitol increase the cell elongation which leads to their lysis and death. They also cause damage to cell membrane. Rifampicin causes inhibition of bacterial DNA dependent RNA synthesis by inhibiting RNA polymerase. Isoniazid inhibits the mycolic acid production, a cell wall component.

Click here to download Figure Figures.docx

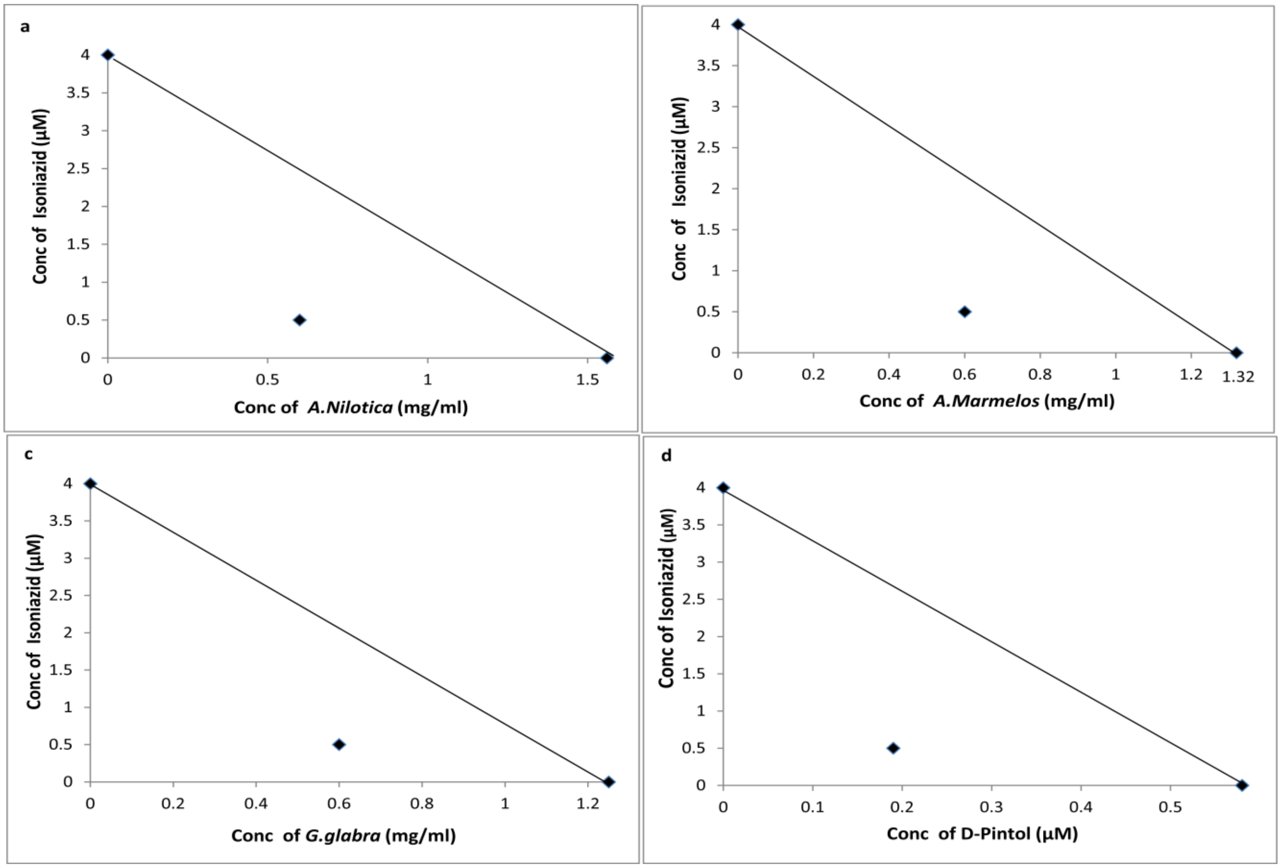

Figure 1. Interaction of Isoniazid with HXM extracts of a) *A. nilotica*, b) *A. marmelos* and c) *G. glabra* and d) the phytochemical, D-Pinitol against *M. smegmatis*. Line indicates additivity. Point below the line indicates synergistic interaction between the two drugs. FIC Index a) 0.33, b) 0.24, c) 0.17, d) 0.21.

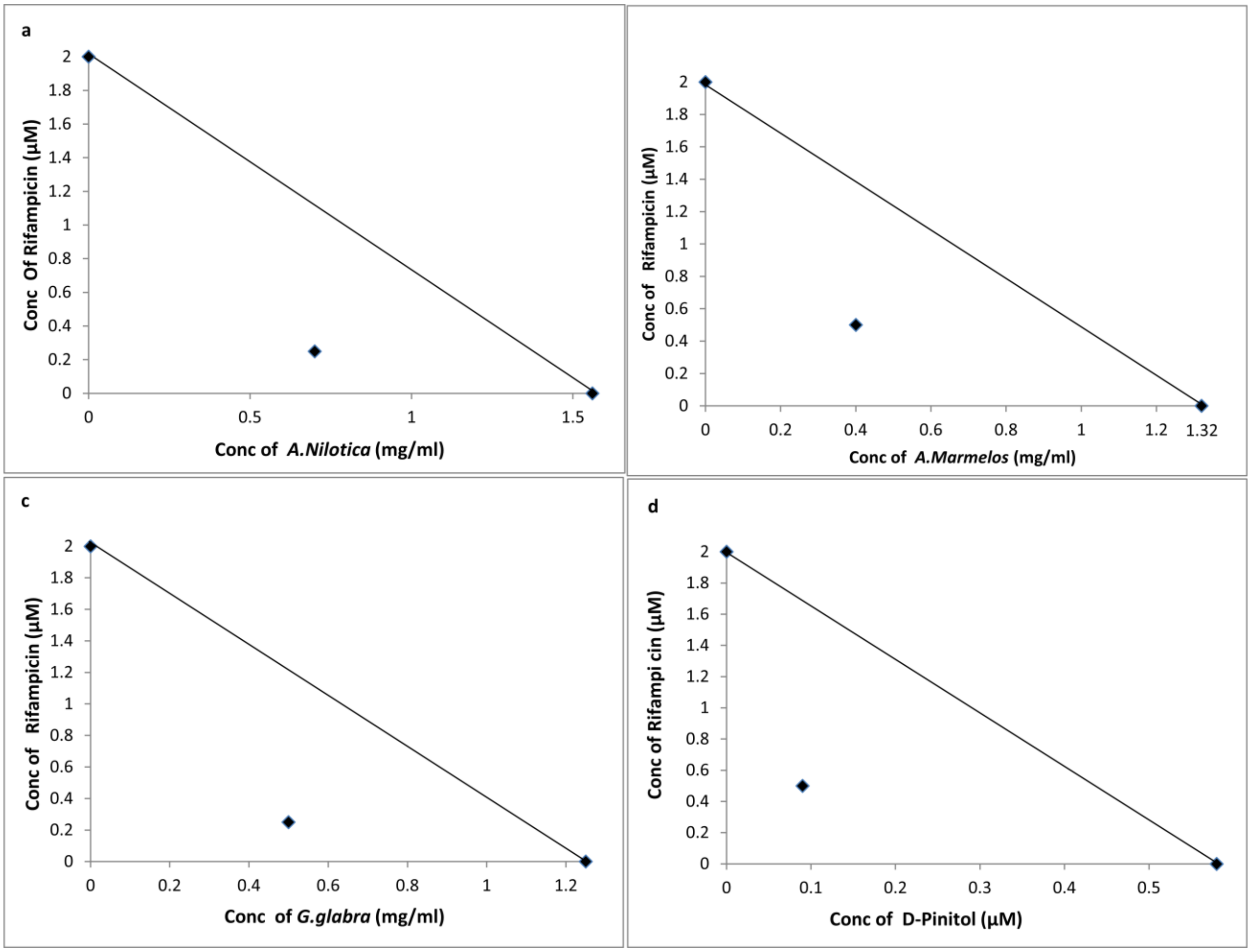

Figure 2. Interaction of Rifampicin with extracts of a) *A. nilotica,* b) *A. marmelos* and c) *G. glabra*, and d) the phytochemical, D-Pinitol against *M. smegmatis.* Line indicates additivity. Points below the line indicates synergistic interaction between the two. FIC Index a) 0.24, b) 0.26, c) 0.15, d) 0.19.

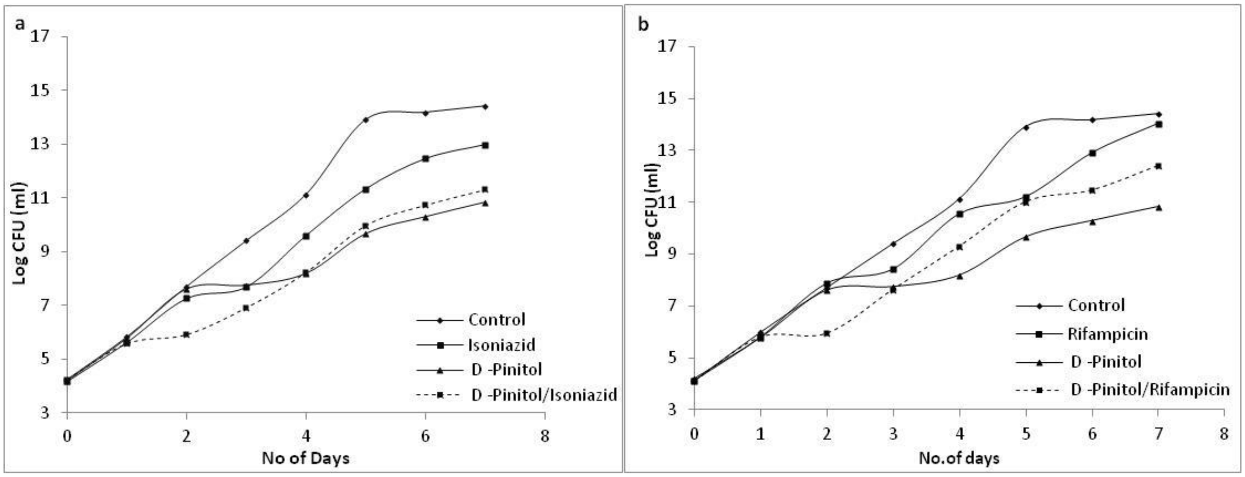

Figure 3. Time Kill curves of a) Isoniazid, b) Rifampicin in combination with the phytochemical D-Pinitol against *M. smegmatis*

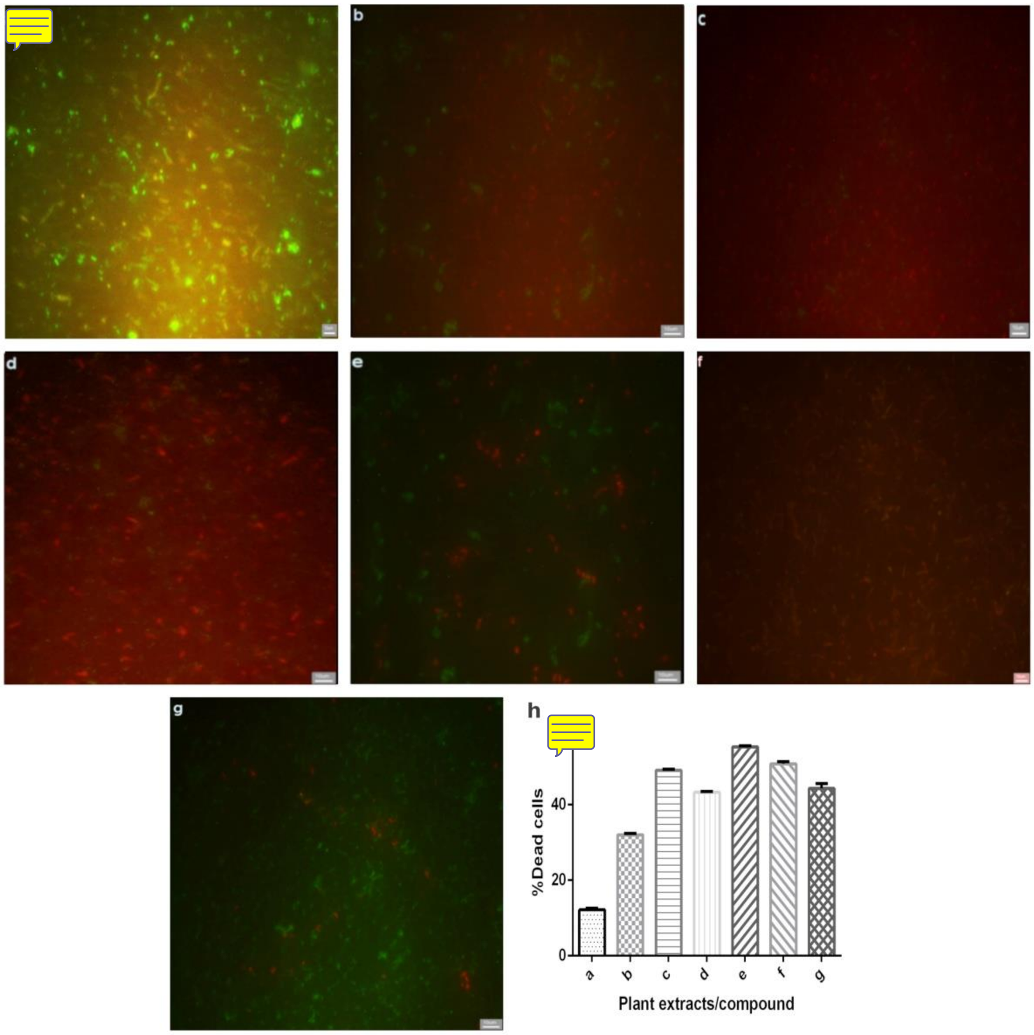

Figure 4. Fluoroscence images of *M. smegmatis* cells on treatment with a) Control, b) Isoniazid, c) Rifampicin, d) *A. marmelos*, e) *A. nilotica*, f) *G. glabra* and g) D-Pinitol [Green indicate viable and Red indicates dead (membrane damaged) cells] h) % of Dead cells on treatment (estimated using Baclight ® dual staining kit).

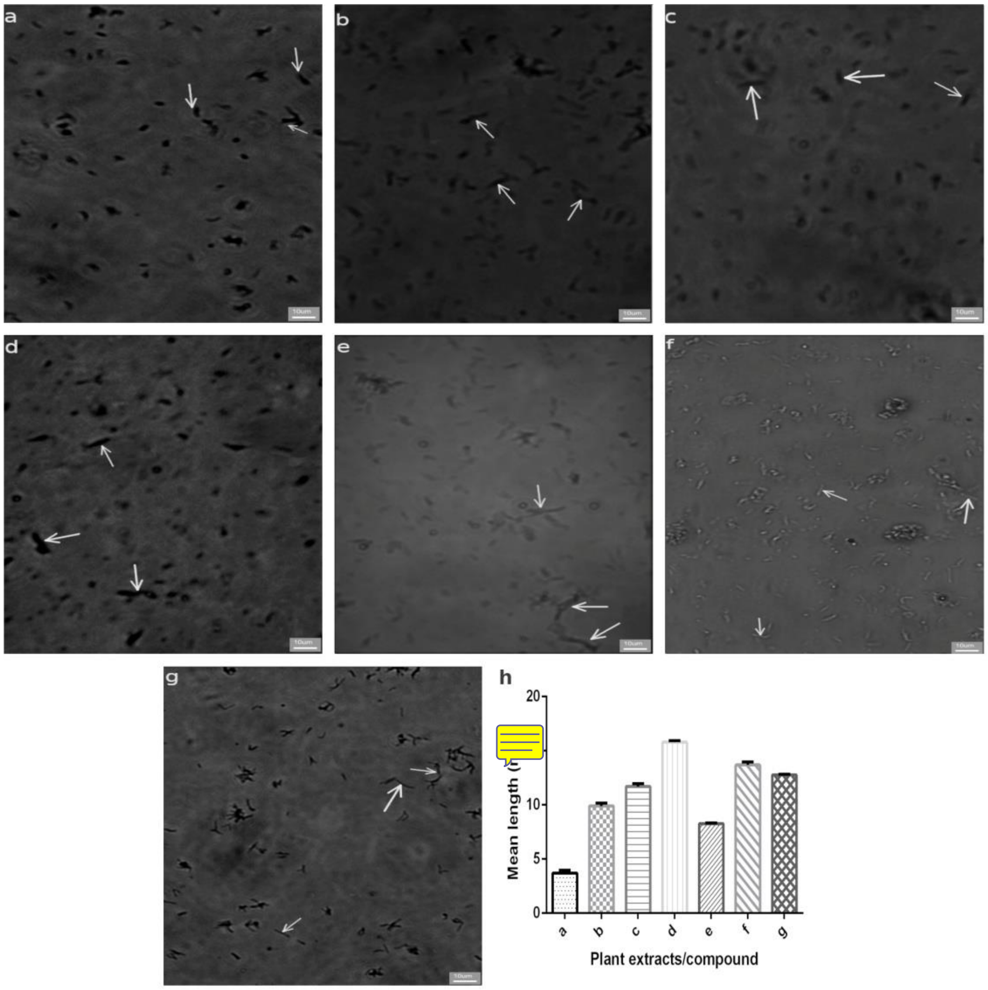

Figure 5. Elongation of *M. smegmatis* cells on treatment with a) Control, b) Isoniazid, c) Rifampicin, d) *A. marmelos*, e) *A. nilotica*, f) *G. glabra* and g) D-Pinitol, h) Average length of *M. smegmatis* cells on treatment. Arrow indicates elongated cells.

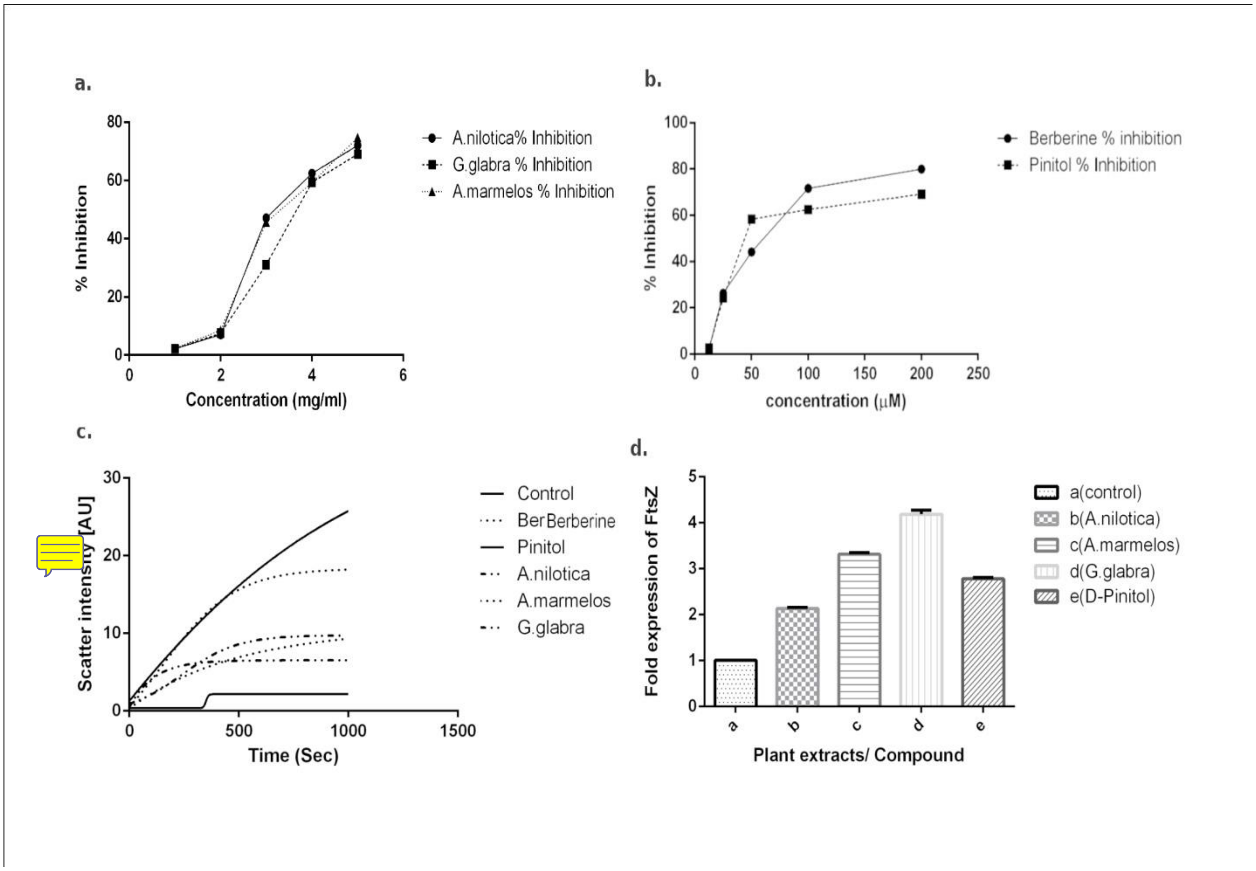

Figure 6. Effect of a) HXM extracts (mg/ml) and b) D-Pinitol (µM) on *M. smegmatis* FtsZ - GTPase polymerisation activity. c) FtsZ polymerisation activity measured using 90° light scattering assay at 1.5 X MIC with FtsZ at 12 µM. Each point represents the mean of three different replicates, d) RT – PCR Gene expression analysis - fold increase in the expression of FtsZ post treatment at 1.5 X MIC.

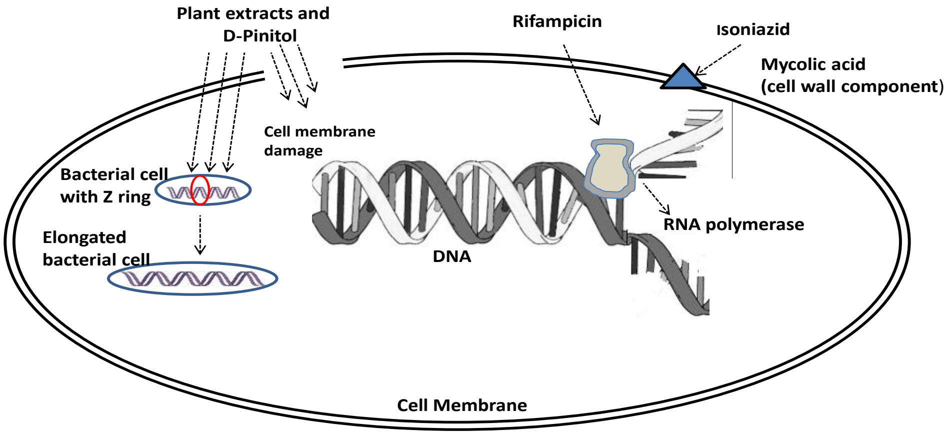

Figure 7. Mechanism of action of HXM extracts, D-Pinitol and commercial drugs on *M. smegmatis* cells. HXM plant extracts and D-Pinitol increase the cell elongation which leads to their lysis and death. They also cause damage to cell membrane. Rifampicin causes inhibition of bacterial DNA dependent RNA synthesis by inhibiting RNA polymerase. Isoniazid inhibits the mycolic acid production, a cell wall component.

Supporting information

Click here to access/download
Supporting Information
Supplementary file.docx
