# Supplementary material for: Inhibitory activity of traditional plants against Mycobacterium smegmatis and their action on Filamenting temperature sensitive mutant Z (FtsZ)—A cell division protein
Source: PLoS One. 2020 May 1;15(5):e0232482. doi: 10.1371/journal.pone.0232482 (PMC7195194; doi:10.1371/journal.pone.0232482)
Supplement: Attachment — Submitted filename: PLOS ONE Response to reviewers.docx [file pone.0232482.s018.docx]

**Comments to the Author

Reviewer #1:** Manuscript Number: PONE-D-19-29098
1, Manuscript Title: Antitubercular activity of plant extracts and their action on cell division protein (FtsZ).The article titled “Antitubercular activity of plant extracts and their action on cell division protein (FtsZ)” talks about possible synergistic effects of plant extracts with anti-mycobacterial drugs which is tested using fast grower M. smegmatis.

The title is quite misleading to any reader with the use of word “anti-tubercular activity”. It is preferred to avoid using the term “anti-tubercular activity” in the title.

**Response:** We have changed the title to“ Inhibitory activity of traditional plants against *Mycobacterium smegmatis* and their action on Filamenting temperature sensitive mutant Z (FtsZ) - a cell division protein”.

2, In line 192, under REMA assay under materials and methods section, RIF and INH is used at 1 mg/ml. This contradicts with several earlier reports for MIC values of Rifampicin and isoniazid which are different as estimated visually or by alamar blue or resazurin assay.

**Response:** The stock of RIF and INH is 1mg/mL in DMSO/ water, from which further 10X dilution was made to avoid DMSO effect. 100 µl from the stock concentration was made up to 1mL with the corresponding DMSO/ water. Such that maximum concentration of 100 µg/mL was used for the assay. We have corrected the above statement under material sections

3, Line 193 under same assay says “The plate was wrapped using aluminium foil and incubated overnight at 37°C for 48 hour”. Kindly clarify if left overnight or for 48 hours. This could be useful to anyone who wants to repeat the assay. Besides, drug treatment for MIC determination would be done more than 24 hours for *M. smegmatis*.

**Response:** It should be written as, the plate was wrapped using aluminium foil and incubated at 37 °C for 48 hour. The term overnight was wrongly placed. We have corrected the above statement under material sections.

4, Line 195-196 under same assay says “The change in colour of the solution from blue to pink indicates inhibition while no change indicates the growth of bacteria”. Actually, the resazurin colour change to pink indicates growth and the statement gives the impression the author(s) is unfamiliar with the protocol.

**Response**: It should be written as the change in color from blue to pink indicates the growth of bacteria and no change indicates inhibition. The above changes were also made in the text file.

5, In line 205, authors refer to synergy drug testing for 24 hours at 37’C and report differential FIC index. However, in Fig 3 detailing time kill analysis we see no difference in CFU as indicated in the graph. What could be the possible explanation for this.

**Response:** In antibacterial study, the MIC was observed post 48 hr. hence there was no significant difference in Time kill at the first 24 hr, however the killing effect (Fig 3) can be seen after 48 hr.

6, In line 214, time kill assay was performed at 1x MIC of the drug. However, routinely synergistic drug effects would be tested at sub MIC levels.

**Response:** Yes, it should be tested under sub MIC levels. But, in synergistic studies, plant extracts acted in subminimal concentration along with the combination of INH/ RIF by reducing the MIC of first line drugs from 4.00 to 0.01 μg/ mL for INH and 2.00 to 0.01 μg/ m for RIF. So,synergy study was performed at lower concentration.

7, In line 271, to study FtsZ gene expression, plant extracts and drugs have been tested at 1.5 x MIC. Is there any reason to use such higher value? Clarity on this will help understand why it was used.

**Response:** The compounds showed antimycobacterial activity against *M.smegmatis* at 1x MIC at 48 hr incubation, but in the case of gene expression studies which is done in 4 hr, the effect of 1x MIC may not show significant difference. Hence in order to mitigate the shorter experiment time, we considered a step higher concentration of 1.5x MIC.

8, In line 282, the authors have written that “There has been no new anti tuberculosis drug introduced in the past 30 years”. Please remove this statement. The authors could have been more careful while writing this.

**Response**: We removed the statement. Thank you for sharing the information on drugs.

9, In line 324, the authors claim that “The results indicated that, these at sub-minimal inhibitory concentration were able to reduce the MIC of both the drugs”. However, the concentration of the HXM extracts used here are given as the same the MIC values given in supplementary table S4. The statement needs clarification.

**Response:** In the study, it is observed that combination of the plant extract with current drugs (RIF/INH) were able to reduce the drug dosages required. Rifampicin and Isoniazid alone exhibited MIC at 2.0 µg/mL and 4.0 µg/mL , whereas in combination with the reported plant extracts they exhibited anti-tubercular activity at 0.01 µg/mL and 0.02 µg/mL. Thus we say that these plant extracts were able to synergize with RIF & INH to exhibit activity at their sub-minimal concentration.

10, In lines 363-365, the authors claim 2-log growth retardation when D-Pinitol was used in combination with Rifampicin or Isoniazid. However, in figure 3, D-pinitol by itself shows better/similar retardation independent of anti-TB drugs. What is the possible explanation of this ??
**Response:** At a concentration of 0.11 µg/mL D- Pinitol showed better MIC activity, whereas first line drug such as Rifampicin, Isoniazid showed MIC of 2.00 and 4.00 µg/ml. This may be the possible reason for D - Pinitol to show its enhanced activity against *Mycobacterium smegmatis*.

**Reviewer #2**: The manuscript is not written in good English. There are many grammatical, topological and convention related errors some of which are mentioned below.

Comments
Minor:
1. Title is incomplete. Title must include the organism worked upon.

**Response**: We have changed the title to “ Inhibitory activity of traditional plants against *Mycobacterium smegmatis* and their action on Filamenting temperature sensitive mutant Z (FtsZ) - a cell division protein”.

2. **Abstract**: Do not include the objective as first line of abstract. It should not start with “To…..”

**Response:** Changes were made in the text

3. Abbreviate the name of the organism/plant wherever its used for the first time in the text.

**Response:** Changes were made in the text

4. Units used in the text are not constant. Some values are represented in mg/ml, ug/ml and some in uM. It is very difficult for the readers to understand and compare.

**Response:** Values are converted to mg/mL, ug/mL throughout the manuscript

5. Line 22: Hexane and not ‘hexame’

**Response**: Change was made in the text

6. Line 39: in vitro should be italicized here as well as throughout the text.

**Response:** In vitro has changed to *In vitro*

7. Line 59: H37RV is incorrect throughout the text. Correct as H37Rv.

**Response**: H37RV is corrected as H37Rv throughout the document.

8. Section 2.1 says 7H9 broth was procured from Sigma and section 2.5 says the same is from BD. Please correct.

**Response:** 7H9 broth was procured from Sigma and was changed in the text file.

9. Line 269: Correct the gene name as 16S rRNA

**Response:** The gene name is corrected as 16S rRNA

10. Line 269: Write Mycobacterium sp. instead of only genus.

**Response**: *M.tuberculosis* genus is added

11. Line 283: ‘strain of existing drug’ does not make sense

**Response**: The sentence were corrected.

12. Line 297: Write observation instead of statement

**Response:** Change was made in the statement.

13. Line 323: Remove the author’s name for reference 51. Just use number.

**Response**: We have removed author name.

**Major:
14. Section 2.10**:
a) If you added plant extract samples after adding 100ul broth, it will change the volume. How did you keep the volume constant?

**Response**: Microplate serial broth-dilution technique was used according to the below reference. Briefly twice the required maximum concentration of the plant extract is added initially, then it is further serially diluted with broth to reach a minimum concentration. To this 100ul of 0.1 O.D culture was added. Thus the volume is kept constant

b) Why did you use 1mg/ml INH and RIF and not test other concentrations?

**Response:** The stock of RIF and INH is 1mg/mL of DMSO, from which further 10X dilution was made to avoid DMSO effect .100 µl from the stock concentration was made up to 1mL with the corresponding DMSO/ water. Such that maximum Concentration of 100 µg/mL was used for the assay which is further serially diluted to obtain the minimum concentration that shows inhibition of growth. We have corrected the above statement under material sections.

c) Did you use medium control without DMSO as a control for INH?

**Response:** Yes, appropriate controls were followed. Only broth as medium control and DMSO also a control

d) All volumes used should be fixed. Do not write about 20ul and so.

**Response:** We corrected the statement

e). Why did you use 1X MIC for time kill assay and 1.5X for gene expression studies. Give reference or explanation for selecting these numbers.

**Response**:

- 1x MIC was used for time kill based on the synergy studies.

**Explanation**: In time-kill the concentrations of plant extracts were used at 1x MIC whereas the concentration of drugs (RIF & INH) was used at sub-minimal inhibitory concentration which was found to be effective in synergy. So, subminimal concentration was used.

- In gene expression studies a higher fold concentrations were employed, since gene expression studies are generally done in lesser time span. Thus 1.5x MIC was considered.

f). **Section 3.2,** Line 307: ‘hexane wash of plants enhances the activity’..did you study unwashed extracts to compare.

**Response:** It should be written as Hexane methanol wash to enhance the activity. We didn’t study the unwashed extracts to compare the efficiency. We corrected the statement in the text file

**g). Section 3.3**: Paragraph 1 results can be represented as a small table for an easy comparison.

**Response:** Table is included as a separate file.
**i). Conclusion**: Include references for mode of action of INH and RIF (line 442-444).

**Response**: References were included for INH and RIF action.

**Reviewer #3:**

1, The phytochemistry that was done to determine the presence and quantity of pinitol is not clear.

**Response**: We have done both GCMS and HPLC for all the three HXM extracts. From HPLC, results of standard D- Pinitol with a retention time at 10.198 min which matches exactly with the retention value of three plants namely *A.nilotica, A.marmelos* and *G.glabra* at 10.187, 10.143 and 10.135 respectively confirming the presence of D-Pinitol. Moreover, mass fragmentation was done for major peak of *A.nilotica* at 5.162 min retension time and *G.glabra* at 5.169 min retension time which showed 90% similarity index for D- Pinitol. *A.marmelos* at 5.160 min retension time showed 92% of similarity index . This major peak of all the three HXM extracts matches with the derivatives of D- Pinitol, pentakis(trimethylsilyl) ether of about 90 %. MS fragmentation reports were also attached in the supplementary file.

The paper is lacking any statistical analysis, although this is mentioned in the M&M section.

**Response**: We included statistical analysis in the data including p value in all the figures as well as one way Anova.

**Response for Line 147**: Yes, we dried the plant samples in an oven overnight at room temperature (37^ο^C). A oven with a fan is used, which blew air through the trays.

**Response for Line 148**: Fruit pulp was macerated by using mortar and pestle without using any solvents.

**Response for Line 166:** Stock solution was prepared 1mg/mL

**Response for line 175:** The concentration of D- Pinitol to run HPLC is 1mg/mL.

In the whole text, values were converted in to mg and µg/mL.

**Response for line 183:** Protocol has been changed

**Response for line 190**: our plant extracts were dissolved at 100mg/mL of 5 % DMSO. We didn’t observe any microprecipitation because the extract dissolved completely.

**Response for line 191:**

The mycobacterium strain was grown in 7H9 medium to an OD of 0.6 and then diluted to attain a cell concentration of 1x10^6^ CFU/ml and added to each well.

**Response for line 197**: Change has been made

**Response for line 224**: Centrifugation done at 10,000 rpm which corresponds to 11200g

**Response for line 249**: Magnification was done at 40X

**Response for line 260**: HXM extract/ D- Pinitol conc is 1.5 x MIC

**Response for line 262:** Malachite green was added as a color reagent

**Response for line 288:** Fragmentation patterns with their similarity index were attached in supplementary file.

**Response for line 294-296:.** Mass spectra data is attached in supplementary file to confirm the presence of D-Pinitol with a similarity index of 90 % in all the three HXM extracts.

**Response for line 307:** It should be written as hexane methanol wash of the plants enhances the activity. (Changes were made in the text).

**Response for line 310**: Values are converted to mg and µg/mL.

**Response for line 312**: Statement should be written as hexane methanol wash of the plant enhances its activity. However, the cited reference states the increase in the activity of hexane extract of *A.marmelos* this is may be due to variation of plant sources, method of preparation of extracts, preparation of media and growth condition of organism. Cited paper used *M.tuberculois* for their experiment. All these factors may influence the MIC values of the extracts.

**Response for line 317:** The reported value is not inhibition, so we deleted the statement in our text.

**Response for line 318:** We modified the text.

**Reviewer 3** : if D – Pinitol is the major constituent in all the 3 plant extracts and show an low /miC of 0.58um(lower than positive control). surely, the extracts will show much better activity.

**Response for line** 346: Yes, we agree with your statement. During combinatorial studies, plant extracts at subminimal concentration i.e., below their MIC values were able to reduce the amount of INH and RIF to 0.01µg/ml to achieve good activity.

# Reviewer 3 : This is low activity (A. nilotica, A. marmelos and G. glabra inhibited the M.tb FtsZ GTPase activity with an 415 IC50 of 22.50 ± 1.25, 23.45 ± 1.12 and 37.9 ± 1.19 mg/ml respectively). For a plant extract to be activity it should fall within ug/ml conc range especially for enzymatic assays and kinetics.

# Response for line 415 : It should be written as IC50 of 1.399, 1.329, 1.564 mg/mL. All biological activity in this paper was performed in mg level and the concentration level used for this experiment is 0.5 to 2.5mg/mL. So it is expected to fall within mg/mL. Since, D- Pinitol is a phytochemical it expressed GTPase activity in µg/mL. Our study is aimed to design a novel plant inhibitor that reduces the mycobacterial resistance to the other first line drugs.

**Reviewer 3** : if the treatment is done at 1.5 times the MIC is expected to see a reduction. An effect should rather be observed at ½, ¼ or 1/8 MIC.

**Response :** In gene expression studies a higher fold concentrations were employed, since gene expression studies are generally done in lesser time span (4 hr). Thus 1.5x MIC was considered for this study. Whereas MIC studies were performed after 48 hrs of incubation.

**Figure 3** : Standard deviations were included.

**Figure 4** : Stastical values were added

**Figure 5**: Stastical values were added and length is changed to µm.

**Figure 6:** IC 50 values were not calculated for light scattering assay.
