# Supplementary material for: Inhibitory activity of traditional plants against Mycobacterium smegmatis and their action on Filamenting temperature sensitive mutant Z (FtsZ)—A cell division protein
Source: PLoS One. 2020 May 1;15(5):e0232482. doi: 10.1371/journal.pone.0232482 (PMC7195194; doi:10.1371/journal.pone.0232482)
Supplement: Attachment — Submitted filename: RebuttalLetter.docx [file pone.0232482.s019.docx]

**Response to the comments**

**Reviewer #1**: Although the manuscript titled “Inhibitory activity of traditional plants against *Mycobacterium smegmatis* and their action on Filamenting temperature sensitive mutant Z (FtsZ) - a cell division protein” is interesting it is not acceptable for publication in the present version because unless the following concerns are addressed.
**Major concerns** –

**1. Section 3.3 and 3.4** –

**Comments** : The combinatorial effect is performed at 24-hour time point in the present study using resazurin assay. But in time kill studies with usage of 1x MIC of drugs/extracts, no difference is seen between the tested samples at 24 hours.

**Response:** The values of CFU at 24^th^ hour are represented in the table 1. CFU values for combination therapy is lesser than the individual drug/ plant extract. The drugs INH and RIF are bactericidal in nature ( **Ref** **1).** *Mycobacterium smegmatis* doubling time is 6-8 hours **(Ref 2).** So beyond 24 hours, drug or extract are not able to prevent the growth of *M.smegmatis.* It is difficult to differentiate the effect of various treatments after 24 hours in the time kill.

Table 1

| **Treatment** | **CFU - 24^th^ hr** |
| --- | --- |
| Control | 614± 3.35 |
| INH | 522± 4.87 |
| RIF | 502± 3.51 |
| *A.nilotica* | **456±1.89** |
| *A.nilotica*/INH | 416±2.32 |
| *A.nilotica*/RIF | 401±3.21 |
| *A.marmelos* | **445±1.91** |
| *A.marmelos*/INH | 404±1.82 |
| *A.marmelos*/RIF | 411±2.67 |
| *G.glabra* | **434±3.76** |
| *G.glabra* /INH | 391± 4.67 |
| *G.glabra* /RIF | 386±4.56 |
| D-Pinitol | **401±1.96** |
| D-Pinitol/ INH | 294±1.75 |
| D-Pinitol/RIF | 302±2.25 |

**Comments**: No representative figures given for the data of HXM extracts in time kill studies though log differences are mentioned in the text in results section.

**Response:** Representative figures are given in the supplementary data.

**Comments: C**ombination of the drugs and extracts have much less reduction when compared to the extracts or pinitol alone. What is the possible explanation?

**Response:**

- Isoniazid and Rifampicin are bactericidal in nature (**Ref 1**) whereas D-Pinitol and the plant extracts are found to be bacteriostatic. Hence in combination, i.e. plant extract or D- Pinitol in higher concentration and drugs in less concentration might alter the effect. This, variation could be observed significantly after 4 days in the time- kill graphs.
- The above same effect was observed for D- Pinitol/ Pencillin combination against MRSA. In which they observed, D- Pinitol in combination with Pencillin, showed less activity when compared with D- Pinitol at 1X MIC (**Ref 3**).
- The observed decrease in the activity when HXM plant extracts/ D- Pinitol and drugs are combined together  could be due to formation (Mailliard reaction) of adduct between INH/RIF through the amine group with D-Pinitol (sugar). This Mailliard reaction between reducing sugars and amines were reported in studies (**Ref 4**).
- Similar activity observed in plant extracts also. Whereas when D-Pinitol used alone it showed more activity than in combination with other extracts.
- However decrease in potency of antibiotics was observed, particularly when combined with *A.nilotica*/ INH, *A.marmelos*/ INH and *A.marmelos*/ RIF. It was particularly apparent for *G.glabra*, which was able to inhibit the growth of *M.smegmatis*, but enhanced its activity when combined with anti-tuberculosis drugs. (INH/RIF) (**Supplementary Fig 11&12S**).

2. In the cell elongation studies (section 3.6), It would be preferable to count atleast 100 cells and give a graph of different cell lengths as percentage. 2- 3 um, 4-5 um, so on. That would be a better representative of the study of what percentage is increase in cell length rather than a single value.

**Response**: Graph with percentage of cells with different length is provided in **Figure 5h.**

3. In the section 3.7, IC50 value is calculated from linear points and graph for standard compounds looks neither linear or as a dose response. Why were the concentrations chosen as given in the text? They are neither log scale increase nor times MIC. What is the possible reason the authors chose to test them? Is curve fitting done for statistical analysis?

**Response**: Yes, The Curve fitting is done and it is represented in **Figure 6 a, b**. It is done using Graph pad prism. Non linear regression (four parameter model) was performed to obtain the IC50 of the respective treatments.

4. The reason for performing gene expression studies at 1.5X MIC is not scientific. If the authors felt 4 hours is too early then the studies should have been done proper titration of time and x MIC. Otherwise there should be a valid reason authors felt that using 1.5x MIC at 4-hour time point is acceptable. A single point data with no scientific explanation to substantiate the same is not convincing.

**Response:** The effect of cell elongation was observed at 4 hr. Hence, we performed the expression studies also at 4 hr. There are studies in which gene expression is performed in response to 90 min, 3 hr and 5 hr exposure also. For eg: Gene expression is done for *M.tuberculosis* after 3 hr of exposure **(Ref 6)**

**References:**

1. Bacteriostatic and Bactericidal Activity of Antituberculosis Drugs Against Mycobacterium Tuberculosis, Mycobacterium avium-Mycobacterium Intracellulare Complex and Mycobacterium Kansasii in Different Growth Phases, , [S Yamori](https://pubmed.ncbi.nlm.nih.gov/?term=Yamori+S&cauthor_id=1406364) [^1^](https://pubmed.ncbi.nlm.nih.gov/1406364-bacteriostatic-and-bactericidal-activity-of-antituberculosis-drugs-against-mycobacterium-tuberculosis-mycobacterium-avium-mycobacterium-intracellulare-complex-and-mycobacterium-kansasii-in-different-growth-phases/#affiliation-1), [S Ichiyama](https://pubmed.ncbi.nlm.nih.gov/?term=Ichiyama+S&cauthor_id=1406364), [K Shimokata](https://pubmed.ncbi.nlm.nih.gov/?term=Shimokata+K&cauthor_id=1406364), [M Tsukamura](https://pubmed.ncbi.nlm.nih.gov/?term=Tsukamura+M&cauthor_id=1406364), Microbiol Immunol,  36 (4), 361-8,  1992.
2. Adaptation of Mycobacterium smegmatis to Stationary Phase, MARJAN J. SMEULDERS, JACQUIE KEER, RICHARD A. SPEIGHT,† AND HUW D. WILLIAMS, JOURNAL OF BACTERIOLOGY, p. 270–283 Vol(181): 1, 1999.
3. Synergistic effect of (+)-pinitol from Saraca asoca with β-lactam antibiotics and studies on the in silico possible mechanism, [Furkan Ahmad](https://www.researchgate.net/profile/Furkan_Ahmad?_sg%5B0%5D=Wv_BqpATIXevT37mhci5rVTIioNmm4l7bilMCdlX8-3R_cvuW6sqaccGE3WNcRM5HAxarag.uAvyv3pxBlDU52OICwFoik-LPP0GMpJ6ASZkg8x2HzoxixPhMASLXbWfIr3fvGyWYGaL78pzm0yIbdjlR4M7xw&_sg%5B1%5D=j_JBU8ukHq94-m_emkih6nlnHsisRLDIVQwyGYracieFptDrieZI8niOTuM8Gh5tb-e31q4.09ExCl5aNrFjzltTeTIU3bEO9bFo3TycPTCzXV5Xp41SSqn1oMxsF12CZCTFxfD_xKTUpudGgenHqJHyM-6jcw), and [Laxminarain Misra](https://www.researchgate.net/profile/Laxminarain_Misra?_sg%5B0%5D=Wv_BqpATIXevT37mhci5rVTIioNmm4l7bilMCdlX8-3R_cvuW6sqaccGE3WNcRM5HAxarag.uAvyv3pxBlDU52OICwFoik-LPP0GMpJ6ASZkg8x2HzoxixPhMASLXbWfIr3fvGyWYGaL78pzm0yIbdjlR4M7xw&_sg%5B1%5D=j_JBU8ukHq94-m_emkih6nlnHsisRLDIVQwyGYracieFptDrieZI8niOTuM8Gh5tb-e31q4.09ExCl5aNrFjzltTeTIU3bEO9bFo3TycPTCzXV5Xp41SSqn1oMxsF12CZCTFxfD_xKTUpudGgenHqJHyM-6jcw), Journal of Asian Natural Products Research 18(2):1-12(2015).
4. Maillard Reactions in Pharmaceutical Formulations and Human Health, . Int J Pharm Compd, [David W Newton](https://pubmed.ncbi.nlm.nih.gov/?term=Newton+DW&cauthor_id=23696045),  ^,,^15 (1), 32-40, 2011
5. Differential Gene Expression in Auristatin PHE-Treated *Cryptococcus neoformans* Tanja Woyke, Michael E. Berens, Dominique B. Hoelzinger, George R. Pettit, Günther Winkelmann, Robin K. Pettit, ANTIMICROBIAL AGENTS AND CHEMOTHERAPY, , p. 561 567(2004) (Gene expression done at 1.5 x MIC in response to 90 min).
6. Thiol reductive stress induces cellulose-anchored biofilm formation in *Mycobacterium tuberculosis,*[Abhishek Trivedi](https://www.nature.com/articles/ncomms11392#auth-1), [Parminder Singh Mavi](https://www.nature.com/articles/ncomms11392#auth-2), [Deepak Bhatt](https://www.nature.com/articles/ncomms11392#auth-3) & [Ashwani Kumar](https://www.nature.com/articles/ncomms11392#auth-4) , [*Nature Communications*](https://www.nature.com/ncomms) vol 7: 11392, (2016) .(Gene expression done in response to 3 hr)

Reviewer #2: The manuscript still has many grammatical and topological errors. Authors should pay attention to sentence formation as well. I strongly recommend that the authors get the manuscript reviewed by an expert before resubmission.

**Comments:**

Isoniazid and Rifampicin can be abbreviated as INH and RIF respectively throughout the text.

**Response:** Isoniazid and Rifampicin is abbreviated as INH and RIF throughout the text.

**Line 30**: Correct as ‘Time kill kinetic studies indicate..’

**Response**: Corrected as Indicated.

**Line 31:** ‘reduction’ would be better than ‘retardation’

**Response:** Replaced the word.

**Line 41:** ‘in-vitro’ is incorrect. Write ‘in vitro’.

**Response:** Corrected as *in vitro*

**Line 41:** inhibition of FtsZ...

**Response:** inhibition of FtsZ is added

**Line 134-135:** Represent in units and not in weight or volume. Also, mention the method of sterlization for OADC.

**Response:** The mixture was filter sterilized using 0.44µm membrane. It was added separately to the culture medium at 2% concentration (v/v).

**Line 139-141**: Authors probably want to say that ‘they’ did not need any specific permission for collection. It can be written as ‘No specific permission was required for collection of plant parts....’

**Response:** Sentences were corrected

**Line 143:** word ‘species’ is repeated, please remove from the end.

**Response:** the word species is removed.

**Line 151:** Were the dried leaves powdered or crushed? Pls mention in the text.

**Response:** It is mentioned as fine powder.

**Line 155, 195, 212, 216**: Do not write ‘about’ for fixed volumes.

**Response:** About is removed

**Line 157:** How is the mixture undisturbed if it was stirred? Where was it incubated overnight and at what temperature? Pls correct. Write the temperature used for following steps as well.

**Response:** Sentences were corrected.

**Line 162:** Already mentioned in section 1.

**Response:** Line was removed

**Line 177:** Correct as ‘elution was carried out at ….’

**Response:** Sentence has been corrected

**Line 182**: Correct the sentence.

**Response:** Sentence has been corrected.

**Line 193:** Write ‘incubated’ instead of ‘kept’.

**Response:** Replaced the word

**Line 195:** Mention whether 96 well plates used were U-bottom.

**Response:** 96 well plates used were flat bottom

**line 197:** Mention the range of tested concentrations of 3 plant extracts.

**Response:** The range of tested concentration is 0.75 to 100 mg/mL

**Line 203**: 48 hours.

**Response:** corrected as 48 hours.

**Line 204:** ‘resazurin dissolved in water’

**Response:** Sentence was corrected

**Line 204:** ‘after this incubation’ is repeated in the same sentence

**Response:** after this incubation’ is removed

**Line 215**: Mention which cultures.

**Response:** *M.smegmatis* is added

**Line 217**: Correct as ‘plates were further incubated’.

**Response:** The sentence was corrected.

Section 2.10 says that the MIC plates were incubated for 48 hrs while in section 2.11 incubation time is mentioned as 24 hrs. Why is it different for section 2.11? Pls explain.

**Response:** MIC was done at 48 hrs incubation. While, we observed synergy at 24 hrs, since a combination showed better action and it was done at 24 hrs.

**Line 228:** Correct as ‘withdrawn at 0, 1, 2, 3, 4, 5, 6 and 7 days, diluted serially and...’
**Response:** The sentence was corrected.

**Line 229**: Mention time of incubation for the plates.

**Response** : Time of incubation is 48 hours

**Line 235:** Have you used ‘they’ for ‘pellet’. Pls correct.

**Response:** The word pellet is added .

**Line 343:** The concentrations of plant extracts used are exact MICs. How are these sub-minimal?

**Response:** In synergistic studies, plant extracts acted in subminimal concentration along with the combination of INH/ RIF by reducing the MIC of first line drugs from 4.00 to 0.01 μg/ mL for INH and 2.00 to 0.01 μg/ m for RIF.

**Line 375-381:** Where are these results shown in the manuscript? These should be represented as a graph and figure number should be mentioned here.

**Response:** Figure number is included in the manuscript

**Line 401:** Pls explain this estimation.

**Response** : The sentences were corrected.

**Line 419**: Remove ‘an’.

**Response**: The word “an” is removed.
