# Supplementary material for: Inhibitory activity of traditional plants against Mycobacterium smegmatis and their action on Filamenting temperature sensitive mutant Z (FtsZ)—A cell division protein
Source: PLoS One. 2020 May 1;15(5):e0232482. doi: 10.1371/journal.pone.0232482 (PMC7195194; doi:10.1371/journal.pone.0232482)
Supplement: Attachment — Submitted filename: RebuttalLetter.docx [file pone.0232482.s020.docx]

**REBUTTAL LETTER**

**Major comments:**

1. The response to the reviewer’s concerns should be inserted appropriately in the main text of the revised version.  It is not enough to present only in the rebuttal letter. For example, add “*Mycobacterium smegmatis* doubling time is 6-8 hours; So beyond 24 hours, drug or extract is not able to prevent the growth of *M.smegmatis.”* as you mentioned in the rebuttal letter.

**Response:** The above mentioned lines were added in the manuscript.

2. The main component in the plant extracts tested, was deemed to be D-Pinitol, based on RT with the standard. However, no direct evidence has been shown to prove that the component is D-Pinitol.  This limitation should be mentioned in line #367.

**Response:**

- D-Pinitol detection is based on RT in HPLC with the standard. This is now mentioned in this manuscript.
- Also, GC-MS Mass fragmentation was done for peak of *A.nilotica* at 5.162 min retension time and *G.glabra* at 5.169 min retention time which showed 90% similarity index for D- Pinitol. *A.marmelos* at 5.160 min retention time showed 92% of similarity index .
- HPLC on st	andard showed percentage area occupied by D- Pinitol at Retention Time (RT) 10.198 is 100 %.
- HPLC on the three plant HXM extracts *A.nilotica, A.marmelos* and *G.glabra* showed percentage area occupied as 45.12 %, 48.33 % and 55.02 % at RT 10.187, 10.143 and 10.135 respectively which matches very closely with RT of standard D-Pinitol (10.198). (Table 1).
- HPLC – matching of retention time is considered as one of the strongest evidence.
- So based on all these analysis it is concluded the HXM plant extracts has D-Pinitol (Supplementary Figures 7S,8S,9S,10S).

**Table 1: GCMS and HPLCanalysis of D-Pinitol with the retention time and percentage area of the plant extracts (We included this table in main manuscript)**

| **S.NO** | **Name** | **Retension time** | **% Area** |
| --- | --- | --- | --- |
| **GCMS** | | | |
| 1 | *A.nilotica* | 5.162 | 31.83 |
| 2 | *A.marmelos* | 5.160 | 5.86 |
| 3 | *G.glabra* | 5.169 | 10.98 |
| **HPLC** | | | |
| 4 | Standard D-Pinitol | 10.198 | 100.00 |
| 5 | *A.nilotica* | 10.187 | 45.12 |
| 6 | *A.marmelos* | 10.143 | 48.33 |
| 7 | *G.glabra* | 10.135 | 55.02 |

3. There is confusion in Table-1. While in the main manuscript, Table-1 is showing MIC values, in the “response to reviewer” document, Table-1 shows CFU data (not sure if this is log scale or linear). This requires clarification and fixing. Both tables should be presented in the main manuscript.

**Response:** In the manuscript we have now included the CFU data (linear) as Table-3.

4. The data presented in Supplementary table-1 is unclear as to what it represents. The heading should appear as the caption at the top of the table. Plus, the RT and % area seems to be misplaced.  Also, I see peak#15 and 17 as D-pinitol, with different RT and % area.  Same discrepancy between HPLC and GC of each of the plant extracts. These are not consistent with the numbers in the main text (line#312). How do the RT values in the suppl tables of GCMS, correlate with the respective spectrum shown in Suppl-figs-2 to 4 ?. These entities need a full, explanations that should be mentioned in the manuscript.

**Response:**

- The headings are changed to the top of the table. I have checked and RT and % area are not misplaced.
- Peaks 15 shows RT at 4.958 with 10.94 % area and it indicates the presence of D-Pinitol derivatives and its esters with different molecular weights (GCMS library database). Peak 17 of *A.nilotica* shows D-Pinitol at RT 5.162 with 31.83 % area. RT of *A.nilotica* matches with the RT of *A.marmelos* and *G.glabra.*
- The numbers in the main text (line#312) correspond to the RT of D-Pinitol in HPLC reports and they are consistent with the corresponding reports in supplementary figures 7S,8S and 9S.
- To better show the correlation between the spectrum and the respective table, we have now included the original GCMS report as a figure that has both the spectrum as well as the table (Supplementary figures 1S,2S and 3S).
- Explanations on GCMS and HPLC peaks were included in the main manuscript with a table.

5. Section 3.5. mentions that about 50% of cells have “Cell wall damage and presumably cell death”.  How does this observation correlate with the “Cell elongation study”, where it is argued that after treatment with extracts, the cells elongate and fail to replicate and undergo death ?.

**Response:**

- The cell elongation histogram (Figure 5h) shows the percentage of elongated cells, from this it can be observed that more than 50 % of cells were elongated. Comparing these percentages with the cell wall damage percentages, it can be derived that there is a percentage of cells that are both elongated and have cell wall damage. Antibacterial compounds can have different modes of action. Cell wall damage is another mechanism of cell death and there need not be a correlation between cell elongation and cell wall damage.

6. Based on the above point, if M. smeg is killed by cell wall damage and cell elongation during treatment with plant extracts, then how would you justify the increase in CFU of treated bacteria in Fig-3. ?

**Response:**

- Minimum inhibitory concentration (MIC) is defined as the lowest drug concentration that shows 90% growth inhibition of *M. smegmatis* (Ref1 and Ref2). The assay adopted in our research also measures this MIC 90
- In our studies, time kill assay, cell elongation and cellular damage studies were carried out at 1x MIC. At 1x MIC, (as per the definition of MIC) 10 % of the organisms which survive at this specific concentration will acclimatize to this conditions and replicates at further time intervals. Thus, an increase in the CFU of the organism is observed in the graph as a function of time (Figure 3a,b). The same observation has been made in other literature reports as mentioned below:
- γ‑Borono Phosphonate Compounds inhibited 90% of *M smegmatis*  (Ref 3).
- Using Rifampicin at 1 × MIC_90_ , 10% of plated bacteria which survived, started to grow.  With a fractional increase in drug concentration (1.2 × MIC_90_), the number of survivor colonies dramatically fell and growth of colonies were undetectable at 1.4 × MIC_90_ (Ref 4).
- Higher concentration of extracts were not used in our study  since the objective of this study is to find the efficacy of the plant extracts and drugs combinations at lower concentration to inhibit the action of *M.smegmatis*.
- We have included the definition of MIC and the cause for increase in CFU in the manuscript.

**References:**

## 1) P.Fyhrquist,I. Laakso,S. Garcia Marco,[R.Julkunen-Tiitto,](https://www.sciencedirect.com/science/article/pii/S025462991300361X#!)[R.Hiltunen](https://www.sciencedirect.com/science/article/pii/S025462991300361X#!), (2014)Antimycobacterial activity of ellagitannin and ellagic acid derivate rich crude extracts and fractions of five selected species of *Terminalia* used for treatment of infectious diseases in African traditional medicine, [South African Journal of Botany](https://www.sciencedirect.com/science/journal/02546299), (90), 1-16.

2) [Akiho Yagi](https://www.nature.com/articles/ja201723?proof=true&platform=oscar&draft=collection#auth-1), [Ryuji Uchida](https://www.nature.com/articles/ja201723?proof=true&platform=oscar&draft=collection#auth-2), [Hiroshi Hamamoto](https://www.nature.com/articles/ja201723?proof=true&platform=oscar&draft=collection#auth-3), [Kazuhisa Sekimizu](https://www.nature.com/articles/ja201723?proof=true&platform=oscar&draft=collection#auth-4), [Ken-ichi Kimura](https://www.nature.com/articles/ja201723?proof=true&platform=oscar&draft=collection#auth-5), [Hiroshi Tomod](https://www.nature.com/articles/ja201723?proof=true&platform=oscar&draft=collection#auth-6) (2017), Anti-*Mycobacterium* activity of microbial peptides in a silkworm infection model with *Mycobacterium smegmatis,* [*The Journal of Antibiotics*](https://www.nature.com/ja?proof=true&platform=oscar&draft=collection) ,( **70)**, 685–690.

3)Giulia Mancini, Maria Bouda, James M. Gamrat, and John W. Tomsho, (2019), Synthesis and Antimicrobial Evaluation of γ‑Borono PhosphonateCompounds inEscherichia coliandMycobacterium smegmatis , ACSOmega,(4), 14551−14559.

4) [Jun-Hao Zhu](https://www.ncbi.nlm.nih.gov/pubmed/?term=Zhu%20JH%5BAuthor%5D&cauthor=true&cauthor_uid=30310059), [Bi-Wei Wang](https://www.ncbi.nlm.nih.gov/pubmed/?term=Wang%20BW%5BAuthor%5D&cauthor=true&cauthor_uid=30310059), [Miaomiao Pan](https://www.ncbi.nlm.nih.gov/pubmed/?term=Pan%20M%5BAuthor%5D&cauthor=true&cauthor_uid=30310059), [Yu-Na Zeng](https://www.ncbi.nlm.nih.gov/pubmed/?term=Zeng%20YN%5BAuthor%5D&cauthor=true&cauthor_uid=30310059), [Hesper Rego](https://www.ncbi.nlm.nih.gov/pubmed/?term=Rego%20H%5BAuthor%5D&cauthor=true&cauthor_uid=30310059), and [Babak Javid](https://www.ncbi.nlm.nih.gov/pubmed/?term=Javid%20B%5BAuthor%5D&cauthor=true&cauthor_uid=30310059) ,Rifampicin can induce antibiotic tolerance in mycobacteria via paradoxical changes in rpoB transcription, [Nat Commun](https://www.ncbi.nlm.nih.gov/pmc/articles/PMC6181997/). 2018; 9: 4218.

7. The justification to use 1.5x MIC and 4hrs post treatment for gene expression studies should be mentioned in the methods or results section.

**Response:** In our studies, cell elongation and cell damage of the *M.smegmatis* is observed at 4 hours of treatment. Therefore gene expression studies is done at 4 hrs with 1.5 x MIC of plant extracts/ compounds in order to increase the expression of *FtsZ.* Similar to our studies, selected compounds like Piperidine, Bromo di-methoxy coumarin, Di-methyl amino methyl coumarin at the concentration of 1.5 x MIC increased the FtsZ gene expression studies. The justification is now mentioned in section 3.9.

**Reference:**

1. Kartik Mitra, Anju Chada, Mukesh Doble, (2019),Pharmacophore based approach to screen and evaluate novel Mycobacterium cell division inhibitors targeting FtsZ – A modelling and experimental study, [European Journal of Pharmaceutical Sciences](https://www.researchgate.net/journal/0928-0987_European_Journal_of_Pharmaceutical_Sciences), 135(2019)103-112.

**Minor comments:**

8, Lines 369-371. Should be rephrased or removed, since it is speculative. The current study is performed in M. smeg, which is not the causative agent of tuberculosis in humans.

**Response:** Sentence was removed.

9, In the rebuttal letter, Milliard reaction should be corrected to Millard reaction, and the justification should be added in the main manuscript.3e

**Response**: Justification is added in the main manuscript
